# Preferences for formal and traditional sources of childbirth and postnatal care among women in rural Africa: A systematic review

**DOI:** 10.1371/journal.pone.0222110

**Published:** 2019-09-25

**Authors:** Arone Wondwossen Fantaye, Nathali Gunawardena, Sanni Yaya

**Affiliations:** 1 Interdisciplinary School of Health Sciences, University of Ottawa, Ottawa, ON, Canada; 2 Faculty of Medicine, McGill University, Montreal, QC, Canada; 3 The George Institute, University of Oxford, Oxford, England, United Kingdom; Emory University School of Public Health, UNITED STATES

## Abstract

**Background:**

The underutilization of formal, evidence-based maternal health services continues to contribute to poor maternal outcomes among women living in rural Africa. Women’s choice of the type of maternal care they receive strongly influences their utilization of maternal health services. There is therefore a need to understand rural women’s preferred choices to help set priorities for initiatives attempting to make formal maternal care more responsive to women’s needs. The aim of this review was to explore and identify women’s preferences for different sources of childbirth and postnatal care and the factors that contribute to these preferences.

**Methods:**

A systematic literature search was conducted using the Ovid Medline, Embase, CINAHL, and Global Health databases. Thirty-seven studies that elicited women’s preferences for childbirth and postnatal care using qualitative methods were included in the review. A narrative synthesis was conducted to collate study findings and to report on patterns identified across findings.

**Results:**

During the intrapartum period, preferences varied across communities, with some studies reporting preferences for traditional childbirth with traditional care-takers, and others reporting preferences for a formal facility-based childbirth with health professionals. During the postpartum period, the majority of relevant studies reported a preference for traditional postnatal services involving traditional rituals and customs. The factors that influenced the reported preferences were related to the perceived need for formal or traditional care providers, accessibility to maternal care, and cultural and religious norms.

**Conclusion:**

Review findings identified a variety of preferences for sources of maternal care from intrapartum to postpartum. Future interventions aiming to improve access and utilization of evidence-based maternal healthcare services across rural Africa should first identify major challenges and priority needs of target populations and communities through formative research. Evidence-based services that meet rural women’s specific needs and expectations will increase the utilization of formal care and ultimately improve maternal outcomes across rural Africa.

## Background

As part of the 2030 Agenda for Sustainable Development, the 17 Sustainable Development Goals (SDGs) have begun to guide global development initiatives [1, 2]. Maternal health status has been one of the major targets of many global development initiatives in the past 30 years [2, 3]. Today, it continues to be a significant deterrent to the improvement and development of women’s health and well-being. Maternal healthcare services are key indicators for monitoring the quality of maternal care and the progress of maternal health outcomes in the developing world, particularly in Africa [1, 2]. The use of traditional maternal care services is a major determinant of poor maternal health outcomes, including maternal mortality [3]. According to the World Health Organization (WHO), poor maternal health outcomes can be reduced by the utilization of formal antenatal, childbirth and postnatal services in health facilities with professional health attendants [4]. Despite the positive outcomes associated with formal maternal care, many women in Africa, especially in sub-Saharan Africa, still seek and utilize traditional maternal care services with traditional attendants or undertake self-care at home [5]. High maternal mortality rates in the continent are strongly correlated with women’s choices of traditional sources of maternal health services throughout the continuum of maternal care [3, 5]. The antepartum, intrapartum and postpartum periods in the continuum can all be high-risk periods for maternal mortality [1, 6]. A systematic review on the timing of maternal mortality found that most maternal deaths occur during the intrapartum or postpartum period [6]. Today, there are considerable disparities in the health-seeking behaviors and utilization rates of formal childbirth care and postnatal care (PNC) among women living in Africa, with the lowest rates of utilization belonging to women living in rural areas [7–9].

Factors involved in maternal healthcare utilization and choices for maternal care providers, in terms of setting and type of attendants, can vary between and within African countries [1, 5, 8, 9]. Such choices can have a significant impact on health-seeking behaviors and utilization patterns of formal and traditional maternal health services. Research evidence indicates that goals to providing high-quality and high value maternal care are best achieved through holistic, inclusive, and collaborative women-centered models of care [10]. The provision, allure, and uptake of high-quality women-centered care require the consideration of women’s views, such as their healthcare preferences. Preferences can influence a patient’s adherence to care options and thereby the health outcomes that are experienced, including maternal death [11]. Therefore, insight into women’s preferences for maternal health caregivers and care settings is vital for the provision of care that is reflective of, and responsive to, women’s desires and values [11, 12].

With limited systematic evaluation of women’s preferences for maternal care, there is a need to identify and comprehensively understand rural women’s preferences for maternal care services in rural African populations. Therefore, the aim of this systematic review is to narratively synthesize findings from existing qualitative research in order to explore and identify rural women's preferences for sources of childbirth and postnatal care. This qualitative evidence synthesis also aims to identify the factors that contribute to rural women’s preferences for maternal care during the childbirth and postpartum periods. The review provides comprehensive understandings about what women prefer and need across different populations and healthcare contexts in rural Africa. While the impact of women’s decision to choose traditional care on maternal outcomes is well documented, identifying the factors that could affect their preferences is crucial for building responsive healthcare systems and reducing poor maternal outcomes. Therefore, this review can help to identify the major preferences for sources of maternal care and the contributing factors that may shape expressed preferences across different populations and contexts in rural Africa. Identification of these preferences and the factors that may shape them can help to inform policies and interventions seeking to promote and improve the utilization of formal maternal health services across rural Africa.

## Methods

### Eligibility

The type of reports eligible for this study are full primary research reports of studies conducted in an African country and published in a peer-reviewed journal between 2001 and 2019, in English. This range was selected because the development of the Millennium Development Goals (MDG), first set out in 2001, led to a new wave of research addressing maternal health. Studies published before 2001 were excluded to ensure the review examined recent evidence following the development of MDG 5, which was to improve maternal health. Non-English articles were excluded to avoid linguistic bias in translations. In terms of setting, studies conducted in urban centers or metropolitan areas were excluded. As such, this review included studies that were conducted in the countryside, agricultural settlements, pastoral communities, or nomadic communities outside of urban centers. For studies that did not clearly specify whether their research was conducted in a rural setting, the rurality of study communities was determined by inspecting the grey literature (e.g. government publications) and by emailing the primary authors of the full-text articles being assessed for eligibility.

Qualitative studies that determined the preferences for sources of childbirth care and PNC and the contributing factors among women living in rural areas were eligible. A qualitative approach best gathers a complete representation of women’s preferences, captures nuances missed in quantitative data collection, and provides a comprehensive understanding of the associated factors. Primary studies where the preferences elicited in the findings were either the primary or secondary focus of the research were included. Studies that were based on secondary data analyses were excluded. The qualitative components of mixed methods studies that explored the preferences of rural women were eligible. Commentaries, discussions, reviews, and incomplete primary research reports, as well as studies that were solely quantitative in design were excluded. Studies that only collected the preferences of men, trained attendants, or traditional attendants were excluded, as this review focused on women’s preferences. A prospective review protocol was not registered for this review.

The term ‘preference’ does not have a clear and consistent definition, which is reflected by how it is distinctly conceptualized across disciplines. In economics, preferences can be defined as total subjective comparative evaluations, in which the subject with the choices considers all the options and consequences that affect his or her evaluations [13]. In psychology, preferences can be defined as evaluative judgements in regards to liking or disliking a stimulus, including over other objects or stimuli [14, 15]. In the context of healthcare, there is a convergence in the conceptualization of preferences as the relative desirability of a range of health experiences and care options [11, 16]. As the topic of this review falls into the context of health and medicine, preferences are defined as the relative desirability of formal and traditional maternal care during the intrapartum and postpartum periods.

Consideration and respect of patients’ preferences is the first principle of Picker’s Eight Principles of Patient-Centered Care [17]. In the healthcare context, preferences can be categorized as a construct with various subjective elements. Qualitative research methodologies are a means to explore and analyze patient preferences for treatment options and the reasons for these preferences [18]. However, with the subjective nature of the qualitative research approach and the inherent subjectivity of human perceptions, it is important to recognize that patients’ expressed or reported preferences gathered through qualitative research can differ from their actual preferences. As a result, it is important to note that the maternal care preferences gathered from the included studies can differ from their genuine preferences due to an array of factors, such as interviewer or moderator bias, barriers to financial and physical accessibility, or the inclination to express preferences that resonate with the preferences of a spouse or elders.

#### Search strategy

A systematic search of the peer-reviewed, published literature from 2001–2019 was conducted in May 2019. With the assistance of a librarian, the primary author searched the online databases Ovid Medline, Embase, CINAHL, and Global Health. A range of terms and combinations were used with MeSH terms and/or text words (see **S1 Appendix**). In order to broaden the results, terms relevant to each period in the continuum of care were included. Reference lists of included studies were perused to identify any additional studies that may satisfy the eligibility criteria.

#### Study selection

**Fig 1:**
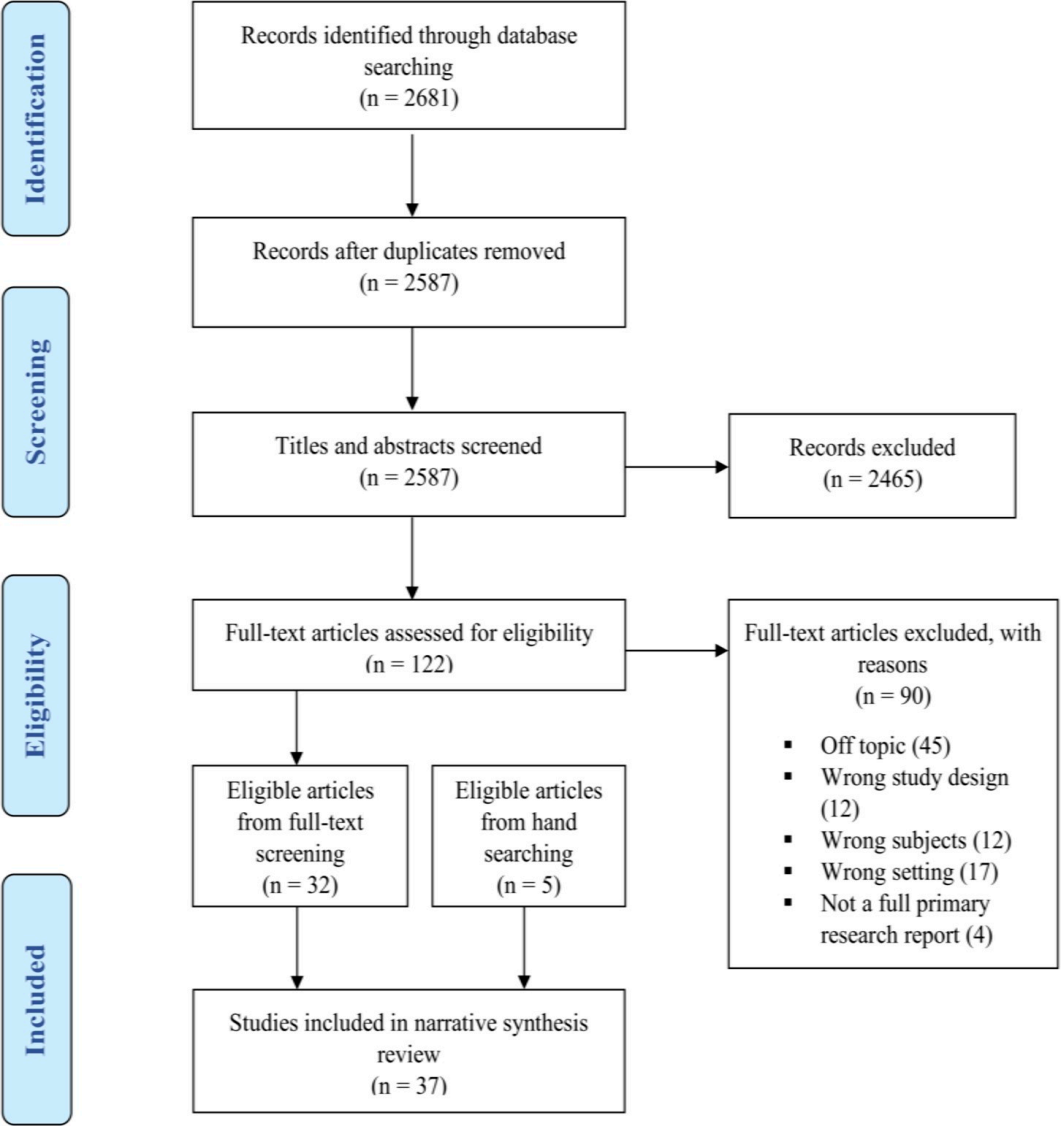
PRISMA flowchart. Selection process for systematic review on rural women’s preferences for maternal care.

The study selection stage, which included screening of titles and abstracts and retrieval of full texts, was carried out independently by AWF and SY (**Fig 1**). The titles and abstracts were screened and then discarded if they did not fit the eligibility criteria. Studies that seemed to include relevant data based on the title and abstract were retrieved, in addition to any unclear citations. The full text versions of the retrieved studies were assessed against the inclusion and exclusion criteria for study eligibility. At each stage, disputes were resolved with discussion. The reference lists of included studies were reviewed, screened and retrieved if eligible for the review, with the process continuing until no new articles were identified.

#### Data extraction

The authors adapted and developed data collection forms based on the needs of the review from a standardized data extraction form by the Cochrane library [19]. The forms ensured data extraction was as consistent as possible across all studies, as the extracted data were used to synthesize the findings. The researchers used the forms to extract the following information from each article: (i) study setting (country); (ii) study aim (s); (iii) sample characteristics; iv) data collection methods; v) main preferences for formal and/or traditional childbirth and/or postnatal care; vi) explanations of why women preferred formal and/or traditional maternal care during the intrapartum and postpartum periods. This review focused on the construct of user preferences across studies exploring women’s maternal care preferences through qualitative methods. Only information of participants and reported preferences relevant to the review were extracted. This made it more feasible to review the selected studies and to synthesize findings. Authors of included studies were contacted through email for additional data, when required.

#### Data synthesis

The narrative synthesis was chosen as the method of synthesis following considerations of time, resources, and appropriateness for addressing the aims of this review. The narrative synthesis adopted in this review was iteratively conducted based on the guidelines for conducting a narrative synthesis by Popay et al. [20]. The method can cope with a large evidence base comprising diverse sources, and effectively address questions that aim to determine or examine an issue. A qualitative narrative synthesis generates a thick text-based description of a phenomenon. The method enables a clear way of integrating and synthesizing primary data findings in a structured manner, helping to generate insights and recommendations directly applicable for policy makers and designers of interventions [21]. It is useful in describing the differences between findings and identifying commonalities within and across groups in a large number of studies [20]. Other methods of qualitative evidence synthesis, including meta-study, meta-narrative, meta-ethnography, grounded theory, and critical interpretive synthesis are more constructivist [21]. They provide a new interpretation of the reviewed phenomenon beyond original data from included studies and are more complex and conceptual. These methods are useful for informing other researchers and theoreticians but require further interpretation by policy makers and designers of interventions [21].

A narrative synthesis is also ideal for identifying research gaps and paths and providing extensive implications for future research [20]. The specific suggestions by Popay et al. [20] as to the tools and techniques appropriate for a narrative synthesis helped enhance the transparency of the qualitative narrative synthesis process and the dependability of the findings and conclusions in this review. The narrative synthesis includes a synthesis of review findings that collates and reports on the findings of included studies in the form of thematic texts. The differences and similarities in reported preferences were then used to combine and analyze evidence in the form of textual summaries and identify relationships within and between studies.

For the synthesis of review findings, texts from the results section of included studies were extracted, including relevant participant quotations, to synthesize the findings on the preferences and corresponding factors. The data on preferences and associated factors were collated into three Microsoft excel spreadsheets corresponding to the categories of formal care, traditional care, and mixed care. Extracted data in these spreadsheets were independently read through thoroughly by AWF and SY to inductively code and identify the salient themes (factors) under which women’s preferences were expressed [22]. AWF and SY agreed that the salient factors were best described by these overarching themes: perceived need of maternal services, accessibility to maternal care, and cultural and religious norms, beliefs, and obligations. The thematic analysis provided the best way to organize and summarize findings in a concise manner from the large body of evidence [20]. The analysis worked with and directly reflected the main ideas and conclusions across included studies rather than developing new knowledge through multiple levels of interpretation. To report the data in a structured and organized manner, the findings were reported in textual format under the major themes [20]. For relationships within studies, differences, similarities and patterns identified within studies by primary authors of included studies were first compiled. Review authors then looked across extracted data to explore and compare relationships across studies [20]. Identified relationships and patterns amongst participant sub-groups, such as by age category, were textually summarized.

#### Quality assessment of included primary studies

The reporting of included studies was assessed using the criteria based on the Critical Appraisal Skills Programme’s (CASP) 10 questions for qualitative research [23]. CASP was selected due to its extensive previous use for systematic reviews of qualitative studies. The domains of the CASP checklist helped assess the credibility and rigor of the included studies and their findings [24]. The 10 questions were designed as prompts to guide reviewers in critically reading the reports. Included studies were assigned an overall score of ‘high’ (9–10), ‘moderate’ (7.5–9) or ‘low’ (less than 7.5) overall quality. Studies were not excluded or weighted based on the quality of the reporting assessment. The results of the qualitative appraisal and assessment (see **S2 Appendix**) were instead used to inform data interpretation and ultimately determine trustworthiness of review findings and conclusions.

#### Assessment of confidence in the synthesis findings

Each qualitative review finding was assessed with the GRADE-CERQual (Confidence in the Evidence from Reviews of Qualitative Research) approach. The method has recently become the standard for assessing confidence in findings from qualitative evidence syntheses and has proven helpful for decision makers and policy designers who use qualitative evidence to inform policies and interventions about various topics, such as healthcare [25]. The CERQual approach assesses the following four concepts: 1) Methodological limitations of included studies; 2) Coherence and fit between data from primary studies and the review findings; 3) Adequacy of data contributing to the review finding; 4) Relevance of the included studies to the context specified in the review question. The factors (sub-themes) that were identified by the authors were assessed for confidence in the methodological quality [26], coherence [27], relevance [28] and adequacy [29] of the contributing data using the GRADE-CERQual tool guidelines for each component.

## Results

### Included studies

Overall, the search across the 4 databases yielded 2681 citations. Of these, 94 duplicates were removed, and 2465 records were excluded after screening titles and abstracts (**Fig 1**). Of the remaining 122 records, 90 were excluded following a full-text review. Thirty-seven reports were included in this review, including 5 additional references from the reference lists of included studies. As shown in **Table 1**, the studies were carried out in 15 different African countries; 1 in Northern Africa (Sudan), 12 in Western Africa, 22 in Eastern Africa, and 2 in Southern Africa according to the United Nations’ Statistics Division [30]. All 37 of the studies elicited data on women’s preferences for sources of childbirth care services, and 11 of the studies on preferences for sources of PNC services. The majority of the studies were conducted in rural communities, while the others were conducted in predominantly rural communities. The qualitative studies and the qualitative components of the mixed studies were primarily based on the use of data collected using focus group discussions and interviews from participants, as shown in **S3 Appendix**. The review includes a diverse sample of rural women of different ages and generations that represent a variety of interests and perceptions. The age group of study participants in the included studies ranged from adolescents to elderly mothers. The rural women also varied in marital status, education level, religious affiliation, parity, and health conditions before or at the time of data collection. **S4 Appendix** fully describes the preferences for sources of childbirth and PNC services and the corresponding factors that may influence these preferences from all 37 studies [31–67].

**Table 1:** Description of included studies.

Study	Study Setting	Sample Characteristics	Main Preferences
Adinew et al. 2018	Ethiopia	68 women who had received clinical ANC service for their most recent childbirth, but no recent facility-based childbirth; 40 women had received some formal education; 45 women were multiparous	Traditional childbirth care at or near home
Adinew & Assefa, 2017	Ethiopia	88 women who gave birth to at least one of their previous children in the health facility within 5 years of data collection but gave birth to their most recent child (within 12 months of data collection) at home; 72 women had some formal education; all were multiparous	Traditional childbirth care at or near home
Ahmed et al. 2018	Mali	26 women (18–40 years) who gave birth 3 months preceding data collection were included in the study; all 26 women were married; none had any formal education; *all 26 women were Muslim; 24 women were multiparous	• Traditional childbirth care at or near home • Formal childbirth care in a health facility
Allou, 2018	Ghana	360 women who had sought the services of traditional birth attendants within 5 years of data collection; 165 women with some formal education; majority were multiparous	Traditional childbirth care at or near home
Al-Mujtaba et al. 2016	Nigeria	57 pregnant ANC attendees, HIV positive women, and young women of childbearing age; 54 married women; 52 women with some formal education; 39 Christian women and 18 Muslim women; most were multiparous	Formal childbirth care in a health facility
Bazzano et al. 2008	Ghana	• 14 older mothers/grandmothers • 45 mothers • 28 case histories from women who had recently given birth	Traditional childbirth care at home
Bedford et al. 2012	Ethiopia	• 30 mothers who had recently delivered (primiparous, multiparous, and grand-multiparous) within 7 months of the study; 14 delivered in a health facility, 14 at home, 1 at a health post, 1 on the roadside • 16 pregnant women (primiparous, multiparous, and grand-multiparous)	• Traditional childbirth care for normal childbirth at or near home • Formal childbirth care in a health facility, especially during complicated childbirth
Caulfield et al. 2016	Kenya	Women who had delivered within 2 years of data collection with a traditional birth attendant, skilled birth attendant, or neither	Traditional childbirth care at or near home
Chea et al. 2018	Kenya	30 HIV-infected women (18–49 years); *majority were married (monogamous); *majority had some formal education; majority were Christian; 12 delivered at home; 18 delivered at a health facility	Formal childbirth care in a health facility
Cofie et al. 2015	Ghana	20 mothers of childbearing age who experienced pregnancy, labor or postnatal complications and mothers whose newborns experienced complications	• Traditional childbirth care at or near home as a first line of care, but facility-based care when complications arise • Formal childbirth and postnatal care in a health facility as a first line of care
Dahlberg et al. 2015	Kenya	• 4 HIV positive mothers and 9 HIV negative mothers of children under 2 years of age; 12 had given birth to their most recent baby in a healthcare facility • Older women (aunts, mothers-in law and grandmothers)	Formal childbirth care in a health facility
De Allegri et al. 2015	Burkina Faso	Women who had recently delivered in a health facility or at home	• Traditional childbirth care at home • Formal childbirth and early postnatal care in a health facility
Dodzo & Mhloyi, 2017	Zimbabwe	108 women of reproductive age (14–49 years); 86 were married; 97 had some formal education	Traditional childbirth and postnatal care at or near home
Engmann et al. 2013	Ghana	85 women who were 27 or more weeks pregnant (18–41 years); 75 women were married; 78 women had some formal education; 75 women were Christian and 10 were Muslims	Formal childbirth care in a health facility
Ganle, 2015	Ghana	94 women (15–45 years) who were pregnant at the time of data collection or who had given birth between January 2011 and May 2012; 64 were married; 37 had some formal education; all 94 women were Muslim	• Traditional childbirth care at or near home • Formal childbirth care in a health facility
Ibrhim et al. 2018	Ethiopia	• 60 women who had children less than 24 months of age; majority were married; majority of the women had no formal education; all women were Muslim; 47 women gave birth at home with a TBA, 13 at a health facility • 48 grandmothers; majority of the grandmothers were married; majority of the grandmothers were uneducated; all grandmothers were Muslim	Traditional childbirth care at or near home
Igboanugo & Martin, 2011	Nigeria	8 pregnant women (24–35 years) who recently accessed maternity services; 2 primigravidas and 6 multigravidas	• Traditional childbirth care at or near home • Formal childbirth care in a health facility
Kea et al. 2018	Ethiopia	18 women who had given birth in the previous 2 years or were pregnant at the time of data collection; *all women were married; most women were Christian	Traditional childbirth care at or near home
King et al. 2015	Ethiopia	33 women (17–49 years); 30 women were married; all women were Muslim; most women were multiparous	• Traditional childbirth care at or near home • Formal childbirth care in a health facility
Kumbani et al. 2013	Malawi	12 mothers (20–32 years) who delivered outside a health facility within 3 months of the study; all were married; 11 had some formal education; 11 were multiparous	Formal childbirth care in a health facility
Kwagala, 2013	Uganda	• *2 young women (15–24 years); *both were married; *both had some formal education; *both were Christian • *3 middle-aged women (25–35 years); all were married; *all had some formal education; *all were Christian • *3 older women (over 36 years); * all were married; *all had some formal education; *all were Christian	• Traditional childbirth and postnatal care at or near home • Formal childbirth and postnatal care in a health facility
Kyomuhendo, 2003	Uganda	Women over 15 years of age; most were married	Traditional childbirth and postnatal care at or near home
Magoma et al. 2010	Tanzania	66 women seeking antenatal care, childbirth care and postnatal care at a health unit	Traditional childbirth and postnatal care at or near home, especially for normal births
Mason et al. 2015	Kenya	• 18 adolescents (15–18 years) • 29 women of childbearing age (15–49 years) • 17 recently or currently pregnant women • 9 mothers of child born with an abnormality	Formal childbirth care in a health facility
Moyer et al. 2014	Ghana	• 35 women with newborn infants • 81 grandmothers who had at least one grandchild within the past year of data collection	• Traditional childbirth care at home • Formal childbirth care in a health facility
Myer & Harrison, 2003	South Africa	• 22 women (17–37 years) seeking antenatal care at a clinic; 14 women were married or in a committed relationship; majority of the women had formal education; 5 primigravidas • 7 women who had syphilis	Formal childbirth care in a health facility
Ndirima et al. 2018	Rwanda	20 women (18–43 years) who had delivered in the district hospital within 10 weeks prior to the start of the study; 10 women were primiparous (3 caesarean sections); 10 women were multiparous (3 caesarean sections)	Formal childbirth care in a health facility
Okafor et al. 2014	Nigeria	25 women (20–42 years) who delivered a baby in the previous 2 years prior to the study; at least 13 women completed some formal education	• Traditional childbirth care in any domestic setting • Formal childbirth and postnatal care in a health facility
Osubor et al. 2006	Nigeria	• Teenage girls (15–19 years); most were Christian • Women of childbearing age (20–49 years) and of parity of not more than 4 children; most women had some formal education; most women were Christian • Women in post-childbearing period (50 years and above); most women had some formal education; most women were Christian	• Traditional childbirth care in a traditional setting • Formal childbirth care in a health facility
Pfeiffer & Mwaipopo, 2013	Tanzania	100 women who delivered at a clinic or with the support of a TBA within 2 months prior to data collection; 49 women were married; 65 women had some formal education; 39 women were multiparous	• Traditional childbirth care at or near home • Traditional childbirth care in a private and confidential environment • Formal childbirth care in a health facility
Seljeskog et al. 2006	Malawi	6 women of *childbearing age who had delivered recently; *all women were married; *All women had some formal education; 3 gave birth at home and 3 at a health facility	• Traditional childbirth and postnatal care at or near home • Formal childbirth care in a health facility
Serizawa et al. 2014	Sudan	6 women (16–40 years) of reproductive age who had given birth within 2–3 years prior to the study; all women were married; none completed any formal education; 2 of the younger women (16–30 years) were primiparous and multiparous; 4 of the older women (30–40 years) were multiparous	Traditional childbirth and postnatal care at or near home
Shiferaw et al. 2013	Ethiopia	8 mothers (15–49 years); most women were married; most women were multiparous	• Traditional childbirth and postnatal care at or near home • Formal childbirth care in a health facility, especially for a complicated childbirth
Sialubanje et al. 2015	Zambia	100 women of reproductive age (15–45 years) who had given birth within 1 year prior to the study; 70 women were married; 93 women had some formal education; 50 were multiparous	• Traditional childbirth care at or near home • Formal childbirth care in a health facility
Sisay et al. 2014	Ethiopia	• 63 grandmothers who had given birth to at least 1 child, who in turn had given birth to at least 1 child; none had any formal education; majority of the women were Christian • 74 women who had any child under 5 years of age; all women were married; majority of the women were Christian • 70 younger women (adolescent girls over 15 years); none were married; all women had some formal education; Majority of the women were Christian	• Traditional childbirth care at home for normal childbirth • Formal childbirth care in a health facility, especially for a complicated childbirth
Thwala et al. 2012	Swaziland	15 women (over 18 years) who had at least 1 child and whose last-born child was 2 years old or less; all women were married; most women had some formal education; *14 women were affiliated with tribal religions and 1 with Catholicism; all were multiparous	• Traditional childbirth care at or near home • Formal childbirth care in a health facility
Wilunda et al. 2014	Uganda	459 women who had delivered in the past 5 years	Traditional childbirth care at or near home

* Additional data retrieved from authors of included studies.

### Quality appraisal

The checklist covers the appropriateness of qualitative research, appropriateness of the research design, ethical considerations and standard conceptions for assessing rigor. Quality assessment helped gather the relative strengths and weaknesses of the body of evidence. As shown in **Tables 2–5** below, 16 studies were of high-quality, 13 studies were of moderate quality, and 8 studies were of low-quality. The quality score of each study corresponded with their degree of rigor, with the high-quality studies generating the most trustworthy findings and being the most rigorous. High quality studies and most moderate studies were dependable, clearly demonstrating that with the same data collection methods, the study could be replicated and yield similar results. Most high-quality and moderate studies corroborated their findings, reflecting the truthfulness of the reported preferences and reasons for preferences of maternal care providers. Studies with higher scores had the most credible results and demonstrated the value and potential impact of research findings locally or internationally. The credibility of the results and the authenticity of research findings to a specific context were relatively low in the lower quality studies. With an average score of 8.22 between the included studies, the overall quality of the included studies was generally moderate; therefore, the evidence used to draw conclusions about preferences in the synthesis can be said to be moderately robust and useful to certain extents for the review’s implications and recommendations. However, due to the diverse nature of participants, various locations of recruitment and data collection, and various factors that may influence review findings, the products of the syntheses should be considered with caution as they are not feasibly transferable to just any rural African populations.

**Table 2:** Summary of quality scores based on 10 CASP checklist questions.

Qualitative studies	Adinew 2018	Adinew 2017	Ahmed et al	Allou	Al-Mujtaba et al	Bazzano et al	Bedford et al	Caulfield et al	Chea et al	Cofie et al
Was there a clear statement of research aims?	1	1	1	1	1	1	1	1	1	1
Is a qualitative methodology appropriate?	1	1	1	1	1	1	1	1	1	1
Was the research design appropriate to address the aims of the research?	0.5	1	1	0.5	0.5	0.5	0.5	0.5	1	1
Was the recruitment strategy appropriate to the aims of the research?	1	1	1	1	1	0	1	0.5	1	1
Was the data collected in a way that addressed the research issue?	1	1	1	0	0.5	0	1	1	1	0.5
Has the relationship between researcher and participants been adequately considered?	1	1	1	0	0.5	0.5	1	0.5	0.5	0.5
Have ethical issues been taken into consideration?	1	1	1	0.5	1	0.5	1	1	1	1
Was the data analysis sufficiently rigorous?	0.5	0.5	1	0	0.5	0	1	1	1	1
Is there a clear statement of findings?	0.5	0.5	1	0.5	1	0.5	1	1	1	1
How valuable is the research?	1	0.5	1	0.5	1	0	1	1	0.5	1
**Overall Quality**	**8.5**	**8.5**	**10**	**5**	**8**	**4**	**9.5**	**8.5**	**9**	**9**

**Table 3:** Summary of quality scores based on 10 CASP checklist questions.

Qualitative studies	Dahlberg et al	De Allegri et al	Dodzo & Mhloyi	Engmann et al	Ganle	Ibrhim	Igboanugo & Martin	Kea et al	King et al
Was there a clear statement of research aims?	1	1	1	1	1	1	1	1	1
Is a qualitative methodology appropriate?	1	1	1	1	1	1	1	1	1
Was the research design appropriate to address the aims of the research?	1	1	0.5	1	1	0.5	1	1	1
Was the recruitment strategy appropriate to the aims of the research?	1	1	1	0	1	0.5	1	1	0
Was the data collected in a way that addressed the research issue?	1	1	1	1	1	1	1	1	0.5
Has the relationship between researcher and participants been adequately considered?	0.5	1	1	0.5	1	0.5	1	0.5	1
Have ethical issues been taken into consideration?	1	0.5	1	1	1	1	1	1	1
Was the data analysis sufficiently rigorous?	1	1	0.5	1	1	0.5	1	1	0.5
Is there a clear statement of findings?	1	1	1	1	1	1	1	1	1
How valuable is the research?	1	1	1	1	1	1	1	1	1
**Overall Quality**	**9.5**	**9.5**	**9**	**8.5**	**10**	**8**	**10**	**9.5**	**8**

**Table 4:** Summary of quality scores based on 10 CASP checklist questions.

Qualitative studies	Kumbani et al	Kwagala	Kyomuhendo	Magoma et al	Mason et al	Moyer et al	Myer & Harrison	Ndirima et al	Okafor et al
Was there a clear statement of research aims?	1	1	1	1	1	1	1	1	1
Is a qualitative methodology appropriate?	1	1	1	1	1	1	1	1	1
Was the research design appropriate to address the aims of the research?	0.5	0.5	0.5	0.5	1	0.5	0.5	0.5	0.5
Was the recruitment strategy appropriate to the aims of the research?	0.5	0.5	0	1	1	1	0	1	1
Was the data collected in a way that addressed the research issue?	1	1	0.5	1	1	1	0.5	0.5	0.5
Has the relationship between researcher and participants been adequately considered?	0.5	0.5	0.5	1	1	0.5	0.5	0.5	0.5
Have ethical issues been taken into consideration?	1	1	0	0.5	1	1	0.5	1	1
Was the data analysis sufficiently rigorous?	1	0.5	0	1	1	0.5	0.5	1	0.5
Is there a clear statement of findings?	0.5	0.5	0.5	1	1	1	0.5	1	1
How valuable is the research?	1	0.5	0.5	1	1	1	1	1	0.5
**Overall Quality**	**8**	**7**	**4.5**	**9**	**10**	**8.5**	**5.5**	**8.5**	**7.5**

**Table 5:** Summary of quality scores based on 10 CASP checklist questions.

Qualitative studies	Osubor et al	Pfeiffer & Mwaipopo	Seljeskog et al	Seriizawa et al	Shiferaw et al	Siaulubanje et al	Sisay et al	Thwala et al	Wilunda et al
Was there a clear statement of research aims?	1	1	1	1	1	1	1	1	1
Is a qualitative methodology appropriate?	1	1	1	1	1	1	1	1	1
Was the research design appropriate to address the aims of the research?	0.5	0.5	0.5	1	0.5	0.5	0.5	1	0.5
Was the recruitment strategy appropriate to the aims of the research?	0	1	0.5	0.5	0	1	1	0.5	1
Was the data collected in a way that addressed the research issue?	1	1	0.5	1	1	1	0.5	1	1
Has the relationship between researcher and participants been adequately considered?	0.5	0.5	0.5	1	0.5	0.5	0.5	0.5	1
Have ethical issues been taken into consideration?	0.5	1	1	1	0.5	1	1	1	1
Was the data analysis sufficiently rigorous?	1	0.5	0.5	1	0.5	1	1	1	1
Is there a clear statement of findings?	1	1	0.5	1	0.5	1	1	1	1
How valuable is the research?	0.5	0.5	1	1	1	1	0.5	1	1
**Overall Quality**	**7**	**8**	**7**	**9.5**	**6.5**	**9**	**8**	**9**	**9.5**

### Evidence synthesis of findings

The data reflected preferences for sources of maternal care during the intrapartum and postpartum periods, along with corresponding factors that contributed to the expressed preferences. The sources of maternal care services generally fell under 2 categories. The first category, formal maternal care, takes place in a healthcare facility (hospitals, health center, or clinics) with the assistance of health care professionals (HCPs), such as doctors, nurses and midwives. The second category, traditional maternal care, takes place at or near home with the assistance of traditional community-based actors (CBAs). These CBAs include the following: traditional birth attendants (TBAs), spiritual attendants, mothers-in-law and relatives, neighbors, or elderly women in the community. The three major themes that best describe the factors contributing to women’s preferences were the following: 1) Perceived need of maternal services from a provider; 2) Accessibility of sources of maternal care; 3) Cultural and religious norms, beliefs, and obligations pertaining to women’s care.

#### Factors contributing to preferences for formal maternal care

Perceived need of formal maternal services: facility-based services were preferred over traditional care services because health facilities had the necessary equipment and supplies required for the provision of maternal care. Waiting times contributed to preferences for private health facilities, which were praised for having shorter queues and providing faster services than public (government) health facilities. Women across rural Africa wanted facility-based care due to confidence in the training and technical ability of HCPs to minimize or prevent health risks and ensure positive maternal and neonatal outcomes. Contrary to HCPs, TBAs and other CBAs were said to be incompetent, unprofessional, and to lack updated skills for managing complications. HCPs were also favored because TBAs lacked referral capacities and were less prompt, thereby increasing the likelihood of poor maternal outcomes. In some communities, male HCPs were specifically favored by women because they were believed to be better trained, more knowledgeable, and emotionally stronger than female HCPs.

Attendant attitudes and behavior were major factors that influenced preferences for facility-based providers of childbirth care. Women across rural Africa preferred to receive childbirth care from facilities that employed caring, considerate and sympathetic HCPs, further expressing that health facilities with cruel, insensitive and degrading attendants increased the odds for negative maternal experiences and outcomes. Welcoming nature of reception staff to service users was also an appealing factor for women who wanted facility-based childbirth care. In some communities, male HCPs were specifically preferred to assist childbirth as they were perceived to be more kind and personable than female HCPs. For others, HCPs from private health facilities were particularly perceived to have more positive attitudes and behaviors, forming better overall interpersonal relationships with their patients. Private health facilities were also favored over public health facilities for better reflecting patient desires and opinions during maternal care provision.

With reference to their own or others’ negative previous experiences with complications, such as excessive bleeding, many women preferred facility-based childbirths. Women with positive previous childbirth experiences, such as successful and uncomplicated deliveries in health facilities, also preferred to seek the same services again. Others who had poor previous birth experiences with a CBA alternatively preferred institutional delivery care. An influential factor that contributed to the preference of facility-based deliveries was fear. Fears were often derived from community experiences largely based on one’s own, or others’ experiences. These fears included fears of dying while giving birth, fears of infection, fears of infecting their child, fears of infecting an inexperienced and untrained TBA, and fears of experiencing complications in a domestic setting under the supervision of untrained CBAs.

Comfort was another factor that explains women’s preferences, especially amongst those who highly valued privacy. Facility-based services that provided privacy were preferred by many women who were concerned about giving birth in open settings and having private parts exposed to strangers. Private health facilities were desirably said to provide women with more control over choices regarding their care than in public facilities. With concerns of privacy heightened during exposure to male HCPs, relatives, or neighbors, such control was very important to women. Female HCPs were particularly favored by some women for better protecting privacy, integrity, and secrecy, as well as being able to build close rapport with laboring women. Some women preferred facility-based care because they were especially comfortable and confident when receiving evidence-based care from experienced HCPs over inexperienced HCPs and interns.

Women also credited their preferences for a facility-based childbirth to the promotion, encouragement of, and sensitization to, the significance of skilled childbirth during facility-based ANC visits. Moreover, knowledge and awareness of their health status during pregnancy also helped make facility-based deliveries the favored choice. While health facility attendants educated and advised women about various maternal health and child health matters, traditional care-takers were often unable to educate and give evidence-based advice to women.

Accessibility to formal maternal care: facility-based care, particularly care provided in government hospitals or other public health facilities, was preferred by some women for being cheaper than other formal maternity care settings in or near their communities. In contrast, others wanted maternal care from private facilities because it was cheaper and more affordable than public facilities in or near their communities. Facility-based childbirth care from HCPs favorably helped women avoid the social pressure of delivering in front of relatives who might judge the progression of, and their behavior during, labor and delivery. Facility deliveries were also favored because they helped women with adverse health conditions, such as HIV, avoid the stigma and discrimination that would have accompanied their health status in a traditional childbirth setting.

Cultural and religious norms, beliefs, and obligations: in some communities, a modern shift in the traditional childbirth norms, owing to increased awareness of the high mortality rates and of the dangers associated with pregnancy and childbirth, shaped some women’s preferences for facility-based childbirth care. Health facilities that respected cultural beliefs and provided culturally sensitive maternal care were favored in several rural populations. Some women preferred to deliver in a formal health setting with mature, female health attendants from their own culture or at least a facility attendant that was familiar with their culture and willing to follow-up patients in the community.

Some women preferred health facilities that respected their religious beliefs and provided religiously sensitive maternal care. Adherence to religious interpretations and obligations was especially important for service users during childbirth. Adherence to religion played a key role in the conditions and circumstances that women desired in health facilities. Muslim women in particular preferred facility-based maternal services from HCPs that respect Muslim women's maternity care needs and enable certain religious practices. Female, Muslim HCPs were deemed the most compatible and thereby the most favored care-takers since they shared the same faith, thus enabling the women to protect the sanctity of their bodies and to follow other religious obligations.

#### Factors contributing to preferences for traditional maternal care

Perceived need of traditional maternal services: across rural Africa, women voiced that they preferred traditional childbirth care due to the poor quality of care in health facilities. Women also preferred traditional births due to long waiting times and lack of immediate childbirth care in health facilities. Many women preferred childbirth care at or near home with TBAs and select other CBAs because they were believed to possess crucial skills for detecting danger signs, identifying the position of the fetus and correcting the position if necessary, providing comprehensive and consistent assistance during and after childbirth, and referring those with labor complications to the health facility when necessary. In addition, TBAs were also perceived to be the most competent care-takers for preventing, curing, or managing medical or transcendent complications that can affect the fetus or mother. TBAs were said to best meet women’s maternal service expectations throughout rural Africa when it came to massaging the laboring woman’s abdomen to facilitate smooth delivery, holding the laboring mother during delivery, and providing constant support and counsel during and after childbirth. Shortages and unavailability of equipment, supplies, and drugs required for adequate maternal care in health facilities also contributed to women’s preferences for traditional births. These women were often required to purchase their own medicine and supplies from pharmacies.

Rude, abusive, insensitive, or deliberately negligent HCPs invigorated women towards traditional births at or near home under the guidance of traditional care-takers or self-care. Many women preferred TBAs or other CBAs to supervise their childbirth because they were more sensitive, caring, hospitable, affectionate, and carried a more positive presence than HCPs. CBAs were said to primarily attend to the mother before discussions about payment, making the women feel that CBAs cared more about women’s welfare than payment.

Trust in traditional care and care-takers contributed to preferences for traditional maternal services. Greater trust in the assistance of TBAs and other CBAs, as well as promotion of traditional homebirths by trusted and revered community members, were particular reasons why childbirth at or near home was desired. In contrast, HCPs were seen as strangers. Their professional integrity was questioned, with accusations that they extorted bribes from clients in exchange for high-quality care. Some simply trusted their own guidance and ability to undergo labor and successfully deliver without any assistance, especially assistance that came from HCPs. Accordingly, trust in one’s own experiences to recognize complications and low perceived susceptibility to negative outcomes fortified preferences for facility-based deliveries. In the early postnatal period, several women preferred to receive traditional PNC at or near home due to greater trust in TBAs, relatives, neighbors or spiritual attendants, compared to HCPs with the unseen baby.

Fear of facility-based services and care-takers was another factor that influenced preferences for traditional care services. Women across rural Africa commonly preferred traditional childbirth care and traditional PNC at or near home due to fears of medical procedures and operations conducted in facilities. There were also fears and speculations sparked by recent cases of maternal death in a health facility and fears that vaginal examinations with HCPs would cause labor retraction and harm the fetus, as well as degrade the laboring women. In a traditional homebirth, vaginal examinations were desirably done when the baby’s head crowned, in a gentle manner. Other expressed fears included: bad fortunes from a facility delivery, ramifications for using non-traditional care, being turned away from a facility for arriving too early while not being actively in labor, and delivering outside of the village premises.

Traditional birthing care at or near home took place in a familiar environment with known people, which was a comfortable and highly preferred birthing environment that many women desired. Health facilities on the other hand were seen as foreign environments that were not as comforting as traditional sources of care. TBAs, relatives and other CBAs were favored over HCPs for taking women’s comfort into account during and after childbirth. Examples provided included the following: freedom to express emotions during labor without restrains; use of warm water instead of the cold water used in health facilities; close care and support; desired birthing positions; respect for privacy; respect for family members or neighbors who wanted to attend. Some women preferred to stay in the community throughout childbirth and puerperium due to discomfort with and an aversion for, young or inexperienced HCPs who held authority over the women in facility settings. They felt passive, helpless and foolish in these situations, and thereby wanted to avoid health facilities, especially if staffed by young or inexperienced HCPs.

Two of the major sub-factors pertaining to comfort were birthing positions and privacy. Traditional care-takers favorably enabled women to deliver in the birthing position of their choice as guided by their instincts and desires without being forced into certain positions. This was especially the case amongst those who preferred kneeling or squatting over the more formal supine positions. A traditional childbirth at or near home was also preferred due to concerns about giving birth in open, crowded rooms and exposing private parts to strangers. A traditional birth favorably enabled women to have control over who was allowed in the room and to cover specific body parts that they wanted to conceal for integrity purposes.

Information, knowledge and awareness were also factors that influenced expressed preferences for traditional maternal care. Inclination towards childbirth care outside of a formal care setting was accredited to the reception of insufficient counsel from HCPs about the significance and benefits of facility-based childbirth during ANC visits. In other communities, some preferred traditional childbirth care because that was the only type of care of which they were aware. This lack of information and resultant lack of knowledge and awareness led some women to prefer what they expected would be a simple childbirth without the need for professional assistance. In some cases, many women who did have information about facility-based childbirth care believed it was only necessary and beneficial for managing childbirth complications that a CBA could not manage. To some women, a facility-based childbirth under the guidance of a skilled attendant did not necessarily guarantee safety from poor outcomes. Likewise, facility-based PNC was deemed to only be necessary when serious complications arose after childbirth.

Accessibility to traditional maternal care: traditional care was preferred at or near home from TBAs, other CBAs, or self-care because it was physically closer and required shorter travel time, if any, to access and have assistance during childbirth and puerperium. Traditional care was the easier and more convenient choice. TBAs, relatives, and other CBAs often lived nearby to service users and were available to provide prompt childbirth assistance at an instant’s notice, even at night. In contrast, HCPs often lived in other communities and were thereby relatively harder to access and unavailable for immediate care. Lack of reliable transportation options, including emergency childbirth ambulances, was another factor that influenced the preference to remain in a domestic setting during childbirth. Additionally, rough topographical conditions and dry weather conditions that impact whether one can reach a health facility, contributed to the preference of a traditional birth around home.

Cheaper costs of staying in the community and receiving affordable assistance from a CBA or opting for self-care was a major factor that influenced rural women’s expressed preferences for traditional childbirth care and PNC at or near home. In contrast, facility-based maternal care required finances for transportation, health services, and/or care supplies. Contrary to a facility delivery, a traditional birth at or near home, such as in a traditional maternity home, did not incur supply and transportation fees. In addition, a traditional birth was favored because of flexible payment time-frames and payment options for services provided by CBAs, such as by means of non-monetary items or social favors.

Traditional maternal care was also preferred since it enabled women to resume and attend to their subsistence activities and multiple household responsibilities, such as caring for children. Opportunity costs that result from health facility attendance further encouraged women to stay at home. To keep their husbands from unfaithfulness, HIV infection and marital and family dysfunction during their prolonged absence, women wanted to stay home throughout intrapartum and postpartum. A traditional birth at or near home was favored over an institutional birth because health facilities were deemed socially restrictive for prohibiting relatives or neighbors from accompanying laboring women into the labor ward. On the other hand, the social permissiveness of CBAs to let relatives and other community members into the delivery room enabled women to receive highly coveted physical, emotional and social support during delivery. The accommodation of relatives and other community members was also desired because it helped women avoid feelings of loneliness.

In several rural communities, a traditional birth with the assistance of a CBA or through self-care was a desired custom that enhanced a mother’s social status and standing within her family and the community. It helped women avoid the stigma attached to a professionally assisted childbirth in a health facility. This included negative labels about laboring women’s weakness for relying on modern care-takers, drugs and equipment. Moreover, some women particularly favored self-cared homebirths because they brought high levels of reverence and recognition as a real or strong woman. Others did not want skilled childbirth assistance from a facility-based source because it was perceived as ill-fated, shameful, and associated with unfaithfulness and deceit about the father of the baby. Skilled, facility based childbirth would be a detriment to a mother’s social status. In terms of gender and age, TBAs were favored because they tended to be female and often older, while facility staff members were often male.

Cultural and religious norms, beliefs, and obligations: in many rural populations, traditional childbirth care and traditional care-takers, namely TBAs, were perceived to be the standard providers, having spanned generations. Childbirth was culturally seen as a natural process that should take place at home following local customs and traditions, while health facilities were mainly treatment centers for abnormal situations. Some women preferred childbirth care and PNC at or near home from CBAs, especially TBAs, spiritual healers, or grandmothers, as they held the role of primary maternal care attendants in the local culture. Others preferred traditional births because they did not want to be seen by health attendants that were strangers to their culture. Cultural practices and beliefs strongly contributed to the preference of traditional maternal care over formal maternal care. In several rural populations, CBAs favorably attended to, supported, or took consideration of valued customs and practices during childbirth and puerperium. Examples of key cultural practices that contributed to preferences for traditional care during intrapartum included the following: ensuring blood loss during delivery is kept within the homestead to protect against bewitchment; customary announcements of a baby’s arrival to the community; application of concoctions to prevent labor complications; application of concoctions to facilitate simple delivery. Examples of key cultural practices that contributed to preferences for traditional care during postpartum included the following: retrieval and burial or aerial fixation of the placenta, often around the woman’s home; performance of postnatal rituals with herbs; application of concoctions to prevent postnatal complications; re-infibulation; clamping the baby’s umbilical cord and applying charcoal powder and herbal extracts to the cord stump; giving a mixture of boiled water, sugar and salt to babies to cleanse their stomachs, ease digestion and boost immunity.

A reason health facilities were not favored was because they did not accept cultural practices or provide culturally sensitive services, some of which were perceived to be important for preventing misfortune on newborns. One key example of the health facilities’ poor appeal is the anger caused by their disposal of placentas against the desires of the women and their families. A traditional birth was also preferred because facility-based deliveries were considered a taboo that brought repercussions to families, including obstetric complications and maternal or infant death. The wish to carry out traditional postnatal customs involving the mother and the newborn also kept women at home after delivery. In populations where the mother and newborn were believed to be vulnerable to witchcraft, women wanted to stay in their own premises for the first 40 days after delivery so they can use traditional customs to fend off witchcraft and evil spirits. Some preferred a traditional PNC because they had to remain in seclusion at home with their baby during postpartum for at least a week after delivery. This tradition went up to three months with twins, which would include the in-house seclusion of the mother, the babies and the placentas. The purpose of such seclusion was to prevent diseases caused by people with the ‘evil eye’ and to give the mother time to recover from delivery in the comfort of her home.

Adherence to religious obligations contributed to the desired provision of services. Religiously sensitive childbirth services at home were desired by some Muslim women due to the significance of protecting the sanctity of the female body in Islam, consuming halal meals, and having a quiet place for prayer. Relative to CBAs, HCPs were less religiously sensitive to some Muslim women’s religious obligations and needs. Complicated births were considered cursed and only religious intervention from a spiritual or traditional attendant throughout the intrapartum period was believed to result in a positive birth outcome. Some believed that irrespective of where one gives birth, complications and maternal death would occur for those being punished for past transgressions. As a result, they wanted homebirth because a facility-based childbirth was considered futile even during complicated situations as only a deity could protect them from maternal death.

#### Traditional and formal maternal care

Perceived need of maternal services: some women voiced a preference for traditional childbirth care as a first line of care for ‘normal’ childbirth, but indicated that their preference shifted for facility-based childbirth care as a second line of care or last resort as soon as a complication arose. Likewise, similar transitional preferences were expressed from normal puerperium to abnormal puerperium, such as following the onset of birth recovery complications. Health facilities were merely treatment centers and believed to be better equipped to handle a complicated childbirth than a traditional provider. There were some women who simply felt that either formal or traditional care were appropriate for providing childbirth care, as birth was believed to be a natural occurrence regardless of the source of care.

The CERQual assessment resulted in final classifications of the overall confidence in each review finding as ‘high’, ‘moderate’, ‘low’, or ‘very low’ [68]. The summary review findings and the CERQual assessments are presented below in **Table 6**. See **S5 Appendix** for overall confidence assessments and explanations for confidence assessments of each finding.

**Table 6:** Summary of narrative synthesis findings.

Review Findings (sub-themes and summaries)	Contributing Studies	CERQual Confidence in the Evidence
**Formal maternal care**		
**Attendant capacity and competence—**Greater training and technical abilities of HCPs in providing maternal care contributed to preferences for formal care.	[31, 33–37, 39–42, 44, 48–50, 53–56, 58, 61, 65, 67]	Moderate confidence
**Availability of resources—**Contrary to traditional care, facility-based services were preferred because of the availability of necessary equipment and supplies.	[35, 40, 42, 54, 58, 61]	Low confidence
**Attendant attitudes and behavior—**Preferences for facilities that employed caring, considerate and sympathetic HCPs, as well as welcoming reception staff.	[36, 37, 40–42, 47, 50, 56, 61]	Low confidence
**Previous experiences—**Positive previous experiences in health facilities and poor previous traditional birth experiences in a domestic setting contributed to preferences for maternal care.	[33, 40, 42, 50, 56, 61, 63]	Moderate confidence
**Fear of complications and death—**Fear of infections, birth complications and death under the guidance of unskilled attendants contributed to preferences for facility-based care.	[31, 47, 53, 57, 67]	Moderate confidence
**Comfort and privacy—**Preferences for facilities that provided the user greater control of their surroundings, including privacy desires.	[33, 40–42, 47]	Moderate confidence
**Knowledge and awareness—**Knowledge and awareness of the significance of skilled maternal care contributed to preferences for formal maternal care.	[36, 44, 48, 61]	Moderate confidence
**Costs and affordability—**Preferences for health facilities that provided cheaper services.	[35, 40]	Very low confidence
**Social pressure—**Preferences for facility-based childbirth because it enabled women to avoid social pressure and stigma during homebirths.	[44, 61]	Low confidence
**Cultural norms—**Shift in cultural norms towards facility deliveries contributed to preferences for formal maternal care.	[48–50, 65]	Moderate confidence
**Religious beliefs and obligations—**Preferences for health facilities that provided religiously sensitive maternal care and respected religious obligations and needs.	[31, 47, 65]	Very low confidence
**Traditional maternal care**		
**Quality of care—**Traditional childbirth care preferred because of the poor quality of facility-based maternal care.	[41, 51, 58]	Low confidence
**Attendant capacity and competence—**TBAs and other CBAs were preferred for being most competent in managing normal childbirths. They were also believed to have greater abilities in detecting, curing and managing complications.	[32, 35, 38, 42, 51, 53, 57, 58, 63, 65, 66]	Moderate confidence
**Availability of resources—**Equipment, supply, and drug shortages, as well as long waiting times in health facilities contributed to preferences for traditional births.	[37, 44, 63]	Low confidence
**Attendant attitudes and behavior—**TBAs and other CBA were preferred for being more affectionate, sensitive, hospitable, and positive than HCPs.	[31, 39, 41–43, 45, 46, 50–53, 57, 62, 63, 65]	Moderate confidence
**Previous experiences—**Traditional births were preferred because of positive previous experiences with traditional births.	[38, 45, 50, 54, 57, 58]	Moderate confidence
**Trust—**Greater trust in CBAs, traditional childbirth care and PNC practices, or self-care, over HCPs and health facilities contributed to preferences for traditional maternal care.	[37, 38, 43, 44, 49, 51, 52, 54, 58, 65, 66]	Moderate confidence
**Fear of medical interventions—**Fear of facility-based services and related consequences of receiving facility-based services contributed to preferences for traditional maternal care.	[32, 37, 42, 46, 48, 55, 59, 60]	Low confidence
**Comforting environment—**Domestic settings were preferred for being more familiar, whereas health facilities were seen as foreign environments. CBAs helped to provide this desired environment by taking consideration of user comfort (e.g. birthing position), while HCPs were adjudged to be less accommodating.	[32, 38, 42–44, 51–54, 57–60, 62, 64–66]	Moderate confidence
**Privacy—**The lack of privacy in health facilities (e.g. exposure of private parts to strangers) contributed to preferences for traditional births. In domestic settings, women possess greater privacy.	[33, 41, 45, 47, 51, 54, 60, 63–65]	High confidence
**Knowledge and awareness—**Lack of knowledge and awareness about maternal health, as well as misconceptions about the significance of formal care for a normal birth and puerperium, shaped some women’s preferences for traditional care.	[31, 32, 34, 37, 44–46, 50, 51, 58, 59, 62, 63, 65, 67]	Moderate confidence
**Shorter distance and convenience—**Traditional births were favored for being closer and more convenient than institutional births.	[31, 32, 34, 38, 45, 50, 51, 54, 57, 63, 66]	High confidence
**Transportation and topographical difficulties—**Lack of transportation options, poor roads, poor terrains and poor conditions contributed to preferences for traditional maternal care.	[32, 34, 37, 50, 63]	Low confidence
**Costs and affordability—**Preferences for traditional births because of cheaper costs (services, transportation, emergencies) and longer repayment time frames than in health facilities.	[31, 32, 35, 37, 39, 41, 45, 46, 50, 60, 63, 66]	High confidence
**Social constraints—**Domestic chores and responsibilities as well as social permissiveness of CBAs in terms of family accommodations during maternal care contributed to preferences to stay away from facility-based care.	[32, 37, 44–46, 51, 53, 58, 59, 65, 66]	Moderate confidence
**Social status—**Preferences for traditional care were also affected by the enhanced social status that comes with traditional care and diminished social status that comes with facility-based care.	[43, 44, 46, 51, 57, 65]	Low confidence
**Cultural norms—**Traditional births were favored because they spanned generations and were considered to be the ‘normal’ type of birth.	[31, 32, 41, 43, 44, 46, 50, 51, 53, 55, 58–60, 65, 67]	High confidence
**Cultural beliefs and obligations—**CBAs provided culturally sensitive care and enabled cultural practices during childbirth and postpartum (e.g. burying the placenta).	[35, 37, 38, 43, 45, 48, 51, 52, 58, 65]	Moderate confidence
**Religious beliefs and obligations—**CBAs favorably provided more religiously sensitive care than HCPs. Beliefs that only God can manage complications also contributed to preferences for traditional maternal care.	[31, 32, 45, 47]	Low confidence
**Traditional and formal maternal care**		
**Necessity of skilled care—**Preferences for traditional childbirth and postnatal care as a first line of care for a ‘normal’ childbirth and puerperium transitioned into preferences for facility-based childbirth and postnatal care as a secondary resort (treatment center) during the onset of complications.	[31, 32, 34, 37, 44, 50, 51, 53, 58, 59, 63, 65, 67]	High confidence

### Relationship within and between studies

According to 6 studies, older women mainly preferred childbirth care at or near home, with or without assistance from a CBA [34, 46, 51, 52, 57, 58]. Likewise, multiparous women commonly preferred traditional maternal care at or near home [36, 43, 49, 52, 53, 57]. These preferences may have been influenced by perceptions of experience with maternity, as well as the need to attend to household tasks and chores. Positive previous childbirth experiences could contribute to perceptions of low susceptibility to complications during subsequent births, leading to minimal desire to use evidence-based, formal maternal care. The greater household responsibilities of multiparous women compared to nulliparous and primiparous women, including caring for multiple children, could contribute to their preferences to stay home for maternal care. In several rural communities, women with some formal education mainly preferred formal childbirth care and PNC under the guidance of HCPs [38, 49, 54, 56, 58, 63]. This may have been due to greater knowledge, awareness, and understanding of the risks of maternity and the significance of professionally trained attendants in reducing poor maternal and neonatal outcomes. Women with some formal education may also have greater employment prospects, income, and ability to seek facility-based care than women without any education.

In 9 studies, married women wanted to receive traditional childbirth care and PNC at or near home in a traditional setting [37, 38, 41–43, 45, 53, 59, 64]. This could have been influenced by reduced decision making power of married women among their nuclear and extended families. Other contributing factors may stem from cultural and religious beliefs about the exposure of married women to strangers in a public facility setting. In 7 studies, women with a history of health complications during previous pregnancies or during data collection of the primary studies preferred skilled institutional deliveries [31, 34–36, 49, 57, 61]. Factors that contributed to these preferences may be the perceived experience of HCPs, and the perception that HCPs and health facilities have a greater ability to manage maternal complications compared to a traditional attendant. Four studies indicated that Muslim women preferred to receive either formal care from HCPs that were sensitive to religious obligations, or traditional care that enabled them to consider religious requirements, such as the sanctity of the female body [33, 41, 47, 49]. However, four studies also indicated that religious norms and beliefs may have minimal influence on the preferred sources of care for some Muslim women, as well as Christian women [36, 42, 49, 59].

## Discussion

### Key findings

This qualitative evidence synthesis identified preferences for both formal and traditional childbirth care, and preferences for traditional postnatal care. The major themes correspond with the parent factors that contributed to women’s intrapartum and postpartum care preferences across rural Africa. As shown in the summary table of review findings, though richer data for traditional maternal care resulted in a greater number of contributing factors, the sub-themes describing the preferences for formal and traditional maternal care were fairly similar. The perceived need of services theme included the necessity and benefits of maternal services offered by a provider. Judgements on the benefits and need of services for positive maternal experiences and outcomes were based on general quality of care, attendant competence and capacity, availability of resources, attendant attitudes and behaviors, previous experiences, fear, trust, comfort, and privacy, as well as knowledge and awareness of maternal risks throughout the continuum of care. The accessibility to services theme included the physical, financial and social accessibility of services provided by a source of maternal care. The cultural and religious norms, beliefs, and obligations theme included norms, obligations and expectations of sensitivity during the provision of maternal care. GRADE-CERQual assessments indicated that the confidence in most of the findings was moderate-to-low. Towards the moderate end of the spectrum, this reflects the quantity of included studies and the range of populations, study contexts, and user viewpoints throughout rural Africa. Towards the low end of the spectrum, this reflects the moderate overall quality of included studies and lack of rich data for some contributing factors, such as the availability of resources.

During the intrapartum period, the promotion of skilled childbirth care during ANC and the perceived high level of competence of HCPs in assisting childbirth and ensuring positive birth outcomes, in contrast to CBAs, strongly contributed to preferences for formal maternal care. In some populations, preferences shifted from traditional care to formal care during the onset of complications, with beliefs that formal care providers (attendants and facilities) were better equipped to manage an abnormal childbirth. The perceived high level of competence of traditional and spiritual attendants in facilitating smooth deliveries and managing health complications strongly contributed to preferences for traditional care. Positive previous experiences with traditional births and the perceived rude, impersonal and neglectful behaviors of HCPs compared with the compassionate and hospitable nature of CBAs, were also factors in preferences for traditional care. Additional factors that contributed to preferences for traditional care included fear of medical operations, comforting and private environments, convenience, cheaper costs, social constraints, social image, norms, and sensitivity to cultural and religious practices. During the postpartum period, the significance of postnatal rituals, perceived competence of CBAs in managing complications, trust in CBAs with the neonate, CBAs’ care for women’s comfort, and easier access to nearby traditional services provision contributed to preferences for traditional PNC at or near home. The perceived high level of competence of HCPs in managing health risks and ensuring full recovery from childbirth was a key factor in preferences for formal PNC. Relationships within studies as identified by primary study authors and between studies by the review authors showed that older women and multiparous women often preferred traditional childbirth care at or near home. This was possibly due to perceptions of lower susceptibility and greater experience to manage their own childbirth without professional assistance. Women who were married preferred traditional maternal care, which may be due to the influence of relatives and elders, or possibly their lack of decision making power in the family unit.

### Review of extant literature

Quantitative studies were excluded from this review due to time, resources, and other pragmatic reasons. Also, most quantitative studies relevant to the review topic did not provide a comprehensive understanding of the factors that contributed to women’s preferences. However, findings from these studies are generally consistent with findings from the review, particularly for childbirth preferences. Surveys of women done in rural Zimbabwe and Gambia, found that the majority wanted professional childbirth assistance in a facility [69, 70]. Some women identified attendants’ access to drug supplies and abilities to handle complications as reasons for preferring skilled care, while others favored TBAs for being more helpful, providing confidentiality and expressing sympathy and respect to patients [70]. In a comparative study of predominantly rural women in Nigeria, women who preferred TBAs or patronized TBAs, accepted statements that TBAs give better attention, are friendlier, desirably pray before conducting deliveries, are located closer to patients, and are more accessible [71]. HCPs were preferred or patronized during abnormal deliveries for having better equipment and training to care for obstetric complications. The factors that influenced women’s preferences for both formal and traditional care-takers in these three studies are very similar and consistent with factors identified in the qualitative evidence synthesis findings. Discrete choice experiments on rural women’s preferences for maternal care in East Africa found that women preferred facilities that provided reliable access to medication and equipment, positive and respectful attendants, good technical quality and highly trained providers over cost, distance and transportation [72, 73]. Some women even prefer to travel to distant facilities with higher quality of services or to receive traditional care at home than to receive low quality services from nearby facilities.

Larson et al. [74] found that medical knowledge and provider treatment as well as interpersonal quality of care were major attractants. In contrast to findings in the other discrete choice experiments [72, 73], access to medical equipment and drugs and privacy were not highly valued. Though precipitate labor was not a prominent factor in this qualitative synthesis, a cross-sectional study of Ethiopian pastoralists found that women preferred traditional births mainly because of labor that progressed fast and gave them no time to reach a health facility [75]. A systematic review of traditional medicine in Saharan Africa found that traditional services were sought and used more than modern care during intrapartum and postpartum due to low costs and alignment with social, cultural, and religious values, as well as discontent with modern care [76]. Studies from various contexts in Asian countries also reported similar findings to those of this review. While some Asian women sought facility-based care because of fears induced from negative previous experiences with obstetric complications [77, 78], others sought traditional care because of fears induced by routine and life-saving operations [79]. Many studies across Asia also found that comfort was a key factor, with women preferring traditional births over institutional births because they were able to give birth in the traditionally desired positions [79, 80]. In Indonesia, a qualitative study found that women still preferred traditional births in a domestic setting because of the physical and social inaccessibility of health facility services [81]. Similar to findings from across rural Africa, homebirths were considered to be more convenient due to distance and the need to manage household chores, such as caring for children. Being away from the family, especially in facilities that restrict family accompaniment, made facility-based deliveries socially inaccessible and undesirable [81].

In addition to comfort, physical accessibility and financial accessibility, a study in Laos found that homebirths were preferred due to the upholding of traditional beliefs and practices [80]. Women with reassurances of a positive health status during ANC visits and with a normal start to labor also wanted traditional sources as a first line of care [80, 82]. However, when complications arose and a normal childbirth turned into an abnormal childbirth, preferences shifted to the second line of care, health facilities. In the postnatal period, a systematic review on traditional maternal practices in Asia found that women tend to stay in a domestic setting because confinement of women was common after childbirth [79]. The reason for this confinement was associated with community perceptions of post-childbirth women as weak, fragile, and vulnerable to illnesses. Other factors that kept women at home included superstitions, magic, traditional medicine and herbs, massaging, and behavioral taboos [79]. Most of the included studies in the review did not explicitly explore the comparative value of facility attributes and measures; however, outright preferences related to physical and financial accessibility in the review conversely indicated that cost, distance and transportation contributed to women’s preferences. Also, access to medication and equipment was not a key factor for women who preferred formal care in review findings. Findings on the influence of good interpersonal and technical quality of care are however consistent with findings from the review. In contrast to factors identified in the qualitative evidence, cultural and religious factors did not greatly affect women’s preferences in the quantitative literature [69–74, 83, 84]. Overall, the quantitative African studies and the studies conducted across Asia, corroborate most review findings that technical quality of care, interpersonal quality of care, previous experiences, fear, comfort, physical access, financial access, and social access contribute to women’s preferences for maternal care.

### Strengths and limitations

The main strength of this review is the systematic identification and synthesis of qualitative evidence from across rural Africa, gathering data on preferences for sources of maternal care from women living in rural African populations. A range of rural women with a variety of demographics, cultures, and communities with different challenges and needs were represented in this synthesis. The search strategy was broad and effective in gathering relevant studies, but the inclusion of all eligible studies in the review meant the inclusion of low quality studies. However, despite some methodological concerns and poor reporting in the lower quality studies, they presented authentic and relevant accounts of perceptions pertaining to the context of this review. The findings of these studies did not markedly contradict those of the moderate and higher quality studies. Inferior scores on the CASP rating could also partially be explained by word limits or other editor suggestions of their journals. Another strength is the GRADE-CERQual transparent assessment of how much confidence readers, including decision-makers and policy-makers, can place on the review findings [25].

Narrative synthesis of qualitative evidence is a relatively young method of qualitative evidence synthesis, with limited reported guidance on how to carry out a qualitative narrative synthesis. As a result, complete transparency is an inherent limitation of the narrative synthesis. Unlike other methods of meta-synthesis, including the meta-ethnography and grounded theory synthesis, the narrative synthesis is not ideal for interpreting evidence and developing explanatory models [20]. Therefore, the reviewers’ interpretation of the findings is not part of the synthesis. Implementation of tools and techniques to collate the evidence and report findings relied on the authors’ discretion of best practice, making it difficult for readers to scrutinize judgements and decisions. As is the nature of qualitative research, researcher discretion of best practice inevitably presents potential for bias. To enhance transparency and display judgements, the narrative synthesis and the tools used for data synthesis were thoroughly described as guided by Popay et al. [20]. Though primary authors of studies were contacted to expand on study findings, additional data on participant characteristics was only collected or still stored and accessible by a few authors. This limits the authenticity and transferability of the identified thematic patterns and relationships between sub-groups across rural Africa. Another limitation identified by the CERQual approach is that majority of the review findings were low to moderate in confidence, with only a few high confidence findings. This can limit the dependability and confirmability of some review findings. Due to drastic anticipated changes in the scope, methodology, and reporting of the review at the outset of the review, an a-priori protocol was not pre-specified and submitted. Despite benefits in avoiding deviation, the absence of an a-priori protocol is a limitation as a-priori protocols help reduce bias in the review process and increase transparency of the evidence synthesis.

Studies published in languages other than English were excluded from the review, which may have introduced a language bias and excluded studies conducted in commonly spoken languages such as French, Arabic, and Swahili. The exclusion of studies conducted in Arabic, for example, may have contributed to the fact that only 1 North African country was included in the review. As some of the studies were conducted over ten years ago, it is possible that the data presented in those studies no longer fully reflect women’s current preferences and needs, thereby limiting the relevance of the findings to future policy and interventions design. Over half the studies were conducted in Ethiopia, Kenya, Nigeria and Ghana, which also limits the authenticity within the review findings and further limits the feasible transferability of review findings and implications throughout rural Africa.

Only a few of the included studies covered perspectives about women’s maternal care preferences after childbirth, limiting the dependability, credibility, and transferability of the PNC preferences and contributing factors. For many women, reported preferences for traditional sources of care could have been supplanted by barriers to their access of evidence-based maternal health services. In other words, their reported or expressed preferences may not be genuine and may have instead been influenced by various restraints. These barriers may have included costs, proximity, transportation, topography, lack of knowledge about available modern services, underdeveloped facilities, low decision-making power in the household and family, relatives’ expectations, and inhibitory traditional or religious obligations. For example, it is plausible that a woman who genuinely wanted a facility-based birth, but was hindered by distance or lack of transportation, may have rather reported a preference for a more convenient homebirth to primary researchers. Therefore, some of the expressed preferences may have been completely confounded by such barriers. Concurrently, it is also important to iterate that preferences for formal care do not always translate to utilization of formal care due to the presence of various deterrents to facility-based care, such as decision-making power. Similarly, relationships identified within and between studies are limited in credibility and dependability due to the lack of sub-group comparisons, absence of participant data, and large variations in preferences and contributing factors.

### Implications of findings

The findings of this review have several implications for policy and the design of interventions. The contextual differences across settings, including differences in preferences between specific groups of women in the same study community, signify the complexity of translating findings into policy and interventions. Those who prefer health facilities and HCPs will be receptive to different and specific contextual initiatives, while those who prefer traditional sources of care will likewise be receptive to different and specific contextual initiatives. This review reveals the necessity of considering specific needs and expectations at the individual and community level to improve the access and quality of formal maternal healthcare services. With the impact of women’s personal experiences and others’ past experiences on preferences and intentions to use a provider in the future, it is crucial to increase the quality and allure of formal care services. Technical and interpersonal quality of care of HCPs had more influence on rural women’s preferences than facility infrastructure or equipment, suggesting that technical and interpersonal quality of care are major care priorities for rural women and crucial for the acceptance of formal maternal care. Poor interpersonal abilities of HCPs can reflect the impact and significance of attendant-service user communications on user perceptions of formal maternal care. A positive, respectful and supportive environment, which many women perceived to have at home with CBAs, need to be traits of formal healthcare settings.

Findings also suggest that promoting, informing and educating women of the significance of facility-based childbirth and early PNC during ANC visits can be vital. Engaging specific maternity needs of rural women, such as needs related to personal comfort and privacy, was shown to be vital for the allure and uptake of formal care. Fundamentally, extensive familiarity, trust, and comfort with CBAs is a complex obstruction that may continue to provide women with alternative traditional options parallel to formal care. Review findings suggest that some women who prefer traditional care can be swayed towards evidence-based care if CBAs are amalgamated with the health care system. In addition to the deterrents of the local sources of formal care, the review implies that many women who prefer out of facility care are also reacting to the pull of traditional care in their premises due to structural restraints and sensitivity to cultural or religious obligations. The review illuminates the complexity of attempting to address strongly ingrained cultural and religious beliefs and practices. It also indicates that the family and sometimes unrelated community members, such as elders, can have a significant influence on women's supposed preferences and decisions regarding the use of specific maternal care services. As a result, the review strongly implicates that improving the allure of formal care to women who prefer traditional care will require more than improvements to the quality of formal health care provision. Issues related to accessibility and culture must also be addressed.

### Recommendations for policy and design of interventions

The review findings call for more holistic, multi-faceted approaches across rural Africa in order to overcome context-specific restraints and design interventions to improve utilization of evidence-based maternal care [85]. Significant, successful, and lasting change in utilization patterns and mortality rates requires these series of interventions to be tailored, integrated, and implemented, both at the individual and community levels:

- This could entail improving access to and quality of health systems, starting with political commitment and investments into improving the competence and skills training of HCPs, as well as the availability of high-quality services. It is also imperative that communities are made aware of the necessity of evidence-based care and risks of traditional care through community-based programs, such as health promotion and education programs. The association of maternal education with access and use of skilled maternity care has been found to be positive [86]. Accordingly, health promotion and education programs should be developed at the community level to educate the community about the positive impact of formal care in reducing maternal health risks. Such programs could help women who prefer traditional childbirth care and PNC because of misconceptions or lack of knowledge and awareness about formal care. This can be especially crucial for populations that view formal care facilities as treatment centers or last resorts for only when complications arise. Formal care needs to be established as the first line of care regardless of perceived normalcies.
- In order to have health facilities and HCPs gain the trust and acceptance of women who prefer traditional care, a patient-centered care approach must be adopted in which patient needs, including characteristics which may influence the outcomes of interest such as socioeconomic position, are prioritized. Creating a positive, supportive and accommodating environment in health facility units that consider the needs of women is a recommended strategic measure that can encourage the uptake of evidence-based services. In accordance, HCPs must be made aware of the huge impact abusive attitudes and behaviors have on the appeal of facility-based childbirth and PNC. Training that targets attitude and behavioral changes of HCPs is recommended in order to create more positive, caring, respectful and hospitable environments in facilities. To help women access health facility services, new or existing interventions should also be designed to mitigate physical and economic inaccessibility. In regions with geographical restraints, governments should attempt to make health facilities with skilled personnel as near as possible and seek to improve road conditions and options for modes of transportation. With costs being potential deterrents to some women’s preferences for facility-based care in several study communities, subsidized programs that remove user fees and finance schemes, such as the establishment of community loan funds, should be designed to ensure that costs of formal maternal health services are manageable.
- With the influence of relatives and community elders on choices of maternal care sources, local decision-makers at the household and community levels should be involved and given a role in the design and implementation of local maternal health interventions. Such influential members can help in increasing understanding and alteration of social norms that lead to the stigmatization of women using formal care. In communities where access to, and utilization of, health facilities is significantly hindered by issues such as household tasks, traditional care at or near home can sometimes be the only option. Home visits throughout the maternal periods from accredited HCPs, particularly if based in the community, could serve as a solution to this problem and help women receive evidence-based care [87].
- In some areas where norms and traditional practices are deeply rooted and unlikely to undergo a modern shift, training and integrating traditional attendants to the health system, possibly under the supervision of accredited HCPs, could enhance their skills and competence in providing maternal health services, while smoothening the transition from traditional care to formal care. Such a method has proven to be successful in Laos, where traditional birth norms experienced in the health facility motivated women to seek formal maternal care in the future [88]. CBAs, namely TBAs, should also be given a strategic role in early referrals, dismissing misconceptions about formal maternal health services, and encouraging social change in the utilization of formal maternal health services. Moreover, initiatives attempting to create more culturally and religiously sensitive maternal services should consider permitting acts that pose no danger to the women or their child; for example, this could include permitting women to take their placenta home or allowing family members to accompany laboring mothers into the labor ward to remove negative feelings that can arise from unfamiliarity or loneliness.

### Future research

There are some research gaps in the qualitative evidence required by policy makers and designers of interventions. Studies in other languages should be explored for additional insight from various regions in Africa. Additionally, to consider perceptions of influential community members that may have a strong influence on women’s decision-making power, future research should triangulate findings on preferences from women with other community members, including TBAs, husbands, and community chiefs. Future reviews could explore this topic with another qualitative synthesis method, such as the meta-ethnography in order to triangulate primary research findings with ordered constructs. This topic could also benefit from the deeper levels of interpretation enabled by the more constructivist qualitative evidence synthesis methods, such as the meta-narrative. Future studies should also examine preferences and contributing factors of maternal care from quantitative studies, including discrete choice experiments. There is currently not enough evidence on preferences for types of maternal care and services provision. Future studies should especially explore the preferences, challenges and priority needs of rural women for the utilization of evidence-based PNC. Future research should also examine factors that can foster positive relationships and communication between CBAs and formal health systems. Lastly, considering the diverse regional and community contexts, existing challenges, needs and priorities throughout the continent, future reviews should assess regional or country-specific variations in user preferences. This will help to determine what aspects of the review findings may be transferable to different contexts and which may not. This review was developed and reported according to the Preferred Reporting Items for Systematic Reviews and Meta-analyses (PRISMA) 2009 checklist (see **S6 Appendix**).

## Conclusion

This review identified that women prefer formal or traditional maternal care due to a wide range of factors. Generally, these factors can be categorized as the perceived necessity and benefits of a provider, accessibility to maternal care services, and cultural or religious norms, beliefs, and obligations. Considering the unique contexts and conditions across Africa and thereby the large and diverse number of populations covered in this review, it is important to iterate that consistency of certain findings does not necessarily mean preferences, influential factors, and individual or community needs are feasibly transferable across rural populations. Likewise, findings that are not consistent with other findings in the review or the literature are not necessarily insignificant in certain contexts. This review exemplifies that there are multiple realities regarding women’s preferred choices for maternal care, as well as corresponding factors that may shape preferences. Therefore, it is important to consider that no magic bullet exists to increase uptake of evidence-based, formal maternal care. To increase utilization of evidence-based maternal health care and to reduce maternal mortality across rural Africa, we need to identify existing resources, understand how members of target communities think about and frame maternal health problems, and examine what they consider as priority needs regarding formal maternal care. Interventions designed with high contextual certainty about target population perceptions, as well as existing challenges and needs, will have a better chance of success.

## Supporting information

S1 Appendix Search strategies. Details of search strategies used in Global Health and OVID Medline.
(DOCX)

S2 Appendix Overall narrative description of the findings of the appraisal. (DOCX)

S3 Appendix Description of included studies. Descriptions of characteristics of included qualitative studies and relevant qualitative data of included mixed-methods studies–expanded version of Table 1.
(DOCX)

S4 Appendix Narrative summary of findings from each study. Includes a table summarizing preferences and factors that influenced preferences.
(DOCX)

S5 Appendix CERQual evidence assessments. (XLSX)

S6 Appendix PRISMA 2009 Checklist. Completed checklist demonstrating adherence to PRISMA guidelines for reporting of systematic reviews.
(DOC)
