# Supplementary material for: Preferences for formal and traditional sources of childbirth and postnatal care among women in rural Africa: A systematic review
Source: PLoS One. 2019 Sep 25;14(9):e0222110. doi: 10.1371/journal.pone.0222110 (PMC6760778; doi:10.1371/journal.pone.0222110)
Supplement: S1 Appendix — Details of search strategies used in Global Health and OVID Medline. (DOCX) [file pone.0222110.s001.docx]

**S1 Appendix: Search Strategies for Global Health and Ovid Medline**

GLOBAL HEALTH

_____________________________________________________________________________________

((("maternal health services" or "maternal care" or "obstetric care" or "obstetric services" or "family planning service*" or (("perinatal" or "antenatal" or "antepartum" or "birth*" or "intrapartum" or "postnatal" or "postpartum") AND service*))) AND (("rural" or "remote area" or "remote region" or "isolated area" or "isolated region" or "secluded area" or "secluded region")) AND (("Africa*" or "Algeria" or "Angola" or "Benin" or "Botswana" or "Burkina Faso" or "Burundi" or "Cameroon" or "Cape Verde" or "Central African Republic" or "Chad" or "Congo" or "Ivory Coast" or "Djibouti" or "Egypt" or "Eritrea" or "Ethiopia" or "Gabon" or "Gambia" or "Ghana" or "Guinea" or "Kenya" or "Lesotho" or "Liberia" or "Libya" or "Madagascar" or "Malawi" or "Mali" or "Mauritania" or "Mauritius" or "Morocco" or "Mozambique" or "Namibia" or "Niger" or "Nigeria" or "Reunion" or "Rwanda" or "Sao Tome and Principe" or "Senegal" or "Seychelles" or "Sierra Leone" or "Somalia" or "South Africa" or "Sudan" or "Swaziland" or "Tanzania" or "Togo" or "Tunisia" or "Uganda" or "Zambia" or "Zimbabwe"))) AND yr:[2001 TO 2019]

OVID MEDLINE

_____________________________________________________________________________________

| 1. exp Maternal Health Services/ |  |
| --- | --- |
| 2. exp parturition/ |  |
| 3. ((obstetric* or maternal or prenatal* or postnatal* or birth* or postpartum or neonatal or midwife* or midwives) adj3 (care or service*)).ti,ab,kw. |  |
| 4. Family Planning Services/ |  |
| 5. (family planning adj3 service*).ti,ab,kw. |  |
| 6. 1 or 2 or 3 or 4 or 5 |  |
| 7. Hospitals, Rural/ |  |
| 8. Rural Health/ |  |
| 9. exp Rural Health Services/ |  |
| 10. Rural Population/ |  |
| 11. rural.ti,ab,kw. |  |
| 12. ((remote* or isolated or secluded or inaccessible) adj3 (area? or region? or territor* or sector? or localit* or dwelling or service* or hospital*)).ti,ab,kw. |  |
| 13. 7 or 8 or 9 or 10 or 11 or 12 |  |
| 14. exp Africa/ |  |
| 15. (Algeria or Angola or Benin or Botswana or Burkina Faso or Burundi or Cameroon or Cape Verde or Central African Republic or Chad or Congo or Ivory Coast or Djibouti or Egypt or Eritrea or Ethiopia or Gabon or Gambia or Ghana or Guinea or Kenya or Lesotho or Liberia or Libya or Madagascar or Malawi).ti,ab,kw. |  |
| 16. (Mali or Mauritania or Mauritius or Morocco or Mozambique or Namibia or Niger or Nigeria or Reunion or Rwanda or Senegal or Seychelles or Sierra Leone or Somalia or South Africa or Sudan or Swaziland or Tanzania or Togo or Tunisia or Uganda or Zambia or Zimbabwe).ti,ab,kw. |  |
| 17. Africa*.ti,ab,kw. |  |
| 18. 14 or 15 or 16 or 17 |  |
| 19. 6 and 13 and 18 |  |
| 20. limit 19 to (english language and yr="2001-Current") |  |
| 21. (addresses or autobiography or bibliography or biography or clinical conference or comment or congresses or consensus development conference or consensus development conference, nih or dataset or dictionary or directory or duplicate publication or editorial or government publications or guideline or interactive tutorial or lectures or legal cases or legislation or letter or meta analysis or news or newspaper article or patient education handout or periodical index or practice guideline or "review" or "scientific integrity review" or systematic reviews or technical report or video-audio media or webcasts).pt. |  |
| 22. 20 not 21 |  |
