# Supplementary material for: Preferences for formal and traditional sources of childbirth and postnatal care among women in rural Africa: A systematic review
Source: PLoS One. 2019 Sep 25;14(9):e0222110. doi: 10.1371/journal.pone.0222110 (PMC6760778; doi:10.1371/journal.pone.0222110)
Supplement: S2 Appendix — (DOCX) [file pone.0222110.s002.docx]

**S2 Appendix: Overall narrative description of the findings of the appraisal**

Though all 37 studies demonstrated that a qualitative approach was appropriate for their studies, only 14 studies clearly demonstrated that their research designs were appropriate for addressing the aims of their research (1). Most studies had an appropriate recruitment strategy for the aims of their research, but commonly failed to indicate why certain participants were the most appropriate to provide access to the type of knowledge sought by the study. Overall, 14 of the studies failed to demonstrate a clear recruitment strategy. Data was sufficiently collected in a way that addressed the research issue in most studies, but some authors did not justify their methods or discuss saturation of data. Majority of the studies have not adequately considered the relationship between researcher(s) and participants as they did not critically examine potential bias and influence during research question formulation and data collection. Ethical issues were taken into consideration in 29 studies, with the rest not clearly demonstrating the maintenance of ethical standards or indicating ethical approval from a committee. Although 21 studies had a sufficiently rigorous data analysis process, only a minority of reports demonstrated critical examination of the role, potential bias and influence of the researcher(s) during analysis and selection of data for presentation. 27 of the included studies had a clear statement of findings, using multiple strategies to establish credibility. The remaining 10 studies did not have a clear statement of findings, predominantly due to inadequate discussions of conflicting evidence and/or of the credibility of study findings. Majority of the studies were valuable, and their findings were applicable to local settings. The average overall quality is 8.22 out 10, which suggests the sum quality of the included studies for this review is moderate.

1. Critical Appraisal Skills Programme [Internet]. CASP qualitative checklist: 10 questions help you make sense of a Qualitative research. Summertown Pavilion; 2018. Available from: [https://casp-uk.net/wp-content/uploads/2018/03/CASP-Qualitative-Checklist-Download.pdf. Accessed 2 June 2018](https://casp-uk.net/wp-content/uploads/2018/03/CASP-Qualitative-Checklist-Download.pdf.%20Accessed%202%20June%202018).
