# Supplementary material for: Preferences for formal and traditional sources of childbirth and postnatal care among women in rural Africa: A systematic review
Source: PLoS One. 2019 Sep 25;14(9):e0222110. doi: 10.1371/journal.pone.0222110 (PMC6760778; doi:10.1371/journal.pone.0222110)
Supplement: S3 Appendix — Descriptions of characteristics of included qualitative studies and relevant qualitative data of included mixed-methods studies–expanded version of Table 1. (DOCX) [file pone.0222110.s003.docx]

**S3 Appendix: Description of Included Studies**

**S3 Table.** Description of included studies

| **Study** | **Aim (s)** | **Study Setting** | **Sample Characteristics** | **Data Collection Methods** | **Main Preferences** |
| --- | --- | --- | --- | --- | --- |
| Adinew et al. 2018 | To explore why some women still give birth at home even after receiving clinical ANC | Ethiopia | 68 women who had received clinical ANC service for their most recent childbirth, but no recent facility-based childbirth; 40 women had received some formal education; 45 women were multiparous | - In-depth interviews - Focus group discussions | Traditional childbirth care at or near home |
| Adinew & Assefa, 2017 | To explore why some women who had previous experience of facility-based delivery gave birth at home for their most recent child | Ethiopia | 88 women who gave birth to at least one of their previous children in the health facility within 5 years of data collection but gave birth to their most recent child (within 12 months of data collection) at home; 72 women had some formal education; all were multiparous | - Focus group discussions - Key informant interviews | Traditional childbirth care at or near home |
| Ahmed et al. 2018 | To understand the sociocultural determinants of assisted  childbirth by nomadic women. | Mali | 26 women (18-40 years) who gave birth 3 months preceding data collection were included in the study; all 26 women were married; none had any formal education; *all 26 women were Muslim; 24 women were multiparous | Semi structured interviews | - Traditional childbirth care at or near home - Formal childbirth care in a health facility |
| Allou 2018 | To determine the factors that influence women’s patronization and preference of TBAs and their services in the Tolon district | Ghana | 360 women who had sought the services of traditional birth attendants within 5 years of data collection; 165 women with some formal education; majority were multiparous | Open-ended questionnaires | Traditional childbirth care at or near home |
| Al-Mujtaba et al. 2016 | To evaluate for and compare and contrast faith-related barriers ANC and PMTCT services utilization among Muslim and Christian women | Nigeria | 57 pregnant ANC attendees, HIV positive women, and young women of childbearing age; 54 married women; 52 women with some formal education;  39 Christian women and 18 Muslim women; most were multiparous | Focus group discussions | Formal childbirth care in a health facility |
| Bazzano et al. 2008 | To examine the social costs of skilled attendance at birth to women | Ghana | - 14 older mothers/grandmothers - 45 mothers - 28 case histories from women who had recently given birth | - In-depth interviews - Semi-structured interviews - Focus group discussions | Traditional childbirth care at home |
| Bedford et al. 2012 | To identify reasons why women who access health facilities and utilise maternal newborn and child health services at other times, do not deliver at health facilities | Ethiopia | - 30 mothers who had recently delivered (primiparous, multiparous, and grand-multiparous) within 7 months of the study; 14 delivered in a health facility, 14 at home, 1 at a health post, 1 on the roadside - 16 pregnant women (primiparous, multiparous, and grand-multiparous) | Semi-structured interviews | - Traditional childbirth care for normal childbirth at or near home - Formal childbirth care in a health facility, especially during complicated childbirth |
| Caulfield et al. 2016 | To investigate the sociodemographic factors and cultural beliefs and practices that influence place of delivery for pastoralist women in Laikipia and Samburu | Kenya | Women who had delivered within 2 years of data collection with a traditional birth attendant, skilled birth attendant, or neither | Focus group discussions | Traditional childbirth care at or near home |
| Chea et al. 2018 | To describe the prevalence and correlates of home delivery among HIV-infected women attending care at a rural public health facility in Kilifi | Kenya | 30 HIV-infected women (18-49 years); *majority were married (monogamous); *majority had some formal education; majority were Christian; 12 delivered at home, 18 at a health facility | Focus group discussions | Formal childbirth care in a health facility |
| Cofie et al. 2015 | To explore how birth location preferences influenced women’s pregnancy and labor experiences, and the resultant impact on their birth outcomes | Ghana | 20 mothers of childbearing age who experienced pregnancy, labor or postnatal complications and mothers whose newborns experienced complications | Semi-structured interviews | - Traditional childbirth care at or near home as a first line of care, but facility-based care when complications arise - Formal childbirth and postnatal care in a health facility as a first line of care |
| Dahlberg et al. 2015 | To understand the individual, family and community factors that influence a woman’s choice of place of childbirth in rural Busia | Kenya | - 4 HIV positive mothers and 9 HIV negative mothers of children under 2 years of age; 12 had given birth to their most recent baby in a healthcare facility - Older women (aunts, mothers-in law and grandmothers) | - In depth interviews - Focus group discussions | Formal childbirth care in a health facility |
| De Allegri et al. 2015 | To explore why some women give birth at home while others give birth in a health facility | Burkina Faso | Women who had recently delivered in a health facility or at home | Open-ended interviews | - Traditional childbirth care at home - Formal childbirth and early postnatal care in a health facility |
| Dodzo & Mhloyi 2017 | - To explore reasons why community deliveries are getting more attractive and being preferred by women | Zimbabwe | 108 women of reproductive age (14-49 years); 86 were married; 97 had some formal education | Focus group discussions | Traditional childbirth and postnatal care at or near home |
| Engmann et al. 2013 | - To explore the beliefs and experiences of pregnant women seeking antenatal care in rural Ghana and to understand the barriers to skilled birth attendants and health facility delivery | Ghana | 85 women who were 27 or more weeks pregnant (18-41 years); 75 women were married; 78 women had some formal education; 75 women were Christian and 10 were Muslims | Semi-structured interviews | Formal childbirth care in a health facility |
| Ganle 2015 | - To explore maternity healthcare needs and care experiences of Muslim women and the barriers to accessing and using maternal health services | Ghana | 94 women (15-45 years) who were pregnant at the time of data collection or who had given birth between January 2011 and May 2012; 64 were married; 37 had some formal education; all 94 women were Muslim | - Focus group discussions - Individual interviews | - Traditional childbirth care at or near home - Formal childbirth care in a health facility |
| Ibrhim et al. 2018 | To explore why women in the pastoralist region of Afar still prefer to give birth at home despite the remarkable improvements made in the accessibility of health facilities | Ethiopia | - 60 women who had children less than 24 months of age; majority were married; majority of the women had no formal education; all women were Muslim; 47 women gave birth at home with a TBA, 13 at a health facility - 48 grandmothers; majority of the grandmothers were married; majority of the grandmothers were uneducated; all grandmothers were Muslim | Focus group discussions | Traditional childbirth care at or near home |
| Igboanugo & Martin 2011 | To identify pregnant women’s perceptions of conventional maternity service provision in the Niger Delta regions | Nigeria | 8 pregnant women (24-35 years) who recently accessed maternity services; 2 primigravidas and 6 multigravidas | Semi-structured interviews | - Traditional childbirth care at or near home - Formal childbirth care in a health facility |
| Kea et al. 2018 | To identify factors influencing the use of maternal health services at the primary health care unit level in Sidama zone | Ethiopia | 18 women who had given birth in the previous 2 years or were pregnant at the time of data collection; *all women were married; most women were Christian | - Focus group discussions - In-depth interviews | Traditional childbirth care at or near home |
| King et al. 2015 | To explore the barriers and facilitators to accessing skilled birth attendance in Afar Region | Ethiopia | 33 women (17-49 years); 30 women were married; all women were Muslim; most women were multiparous | Semi-structured interviews | - Traditional childbirth care at or near home - Formal childbirth care in a health facility |
| Kumbani et al. 2013 | To explore the reasons why women delivered at home without skilled attendance despite receiving antenatal care at a health centre and their perceptions of perinatal care | Malawi | 12 mothers (20-32 years) who delivered outside a health facility within 3 months of the study; all were married; 11 had some formal education; 11 were multiparous | In-depth interviews | Formal childbirth care in a health facility |
| Kwagala 2013 | To examine what factors influence choice of place of delivery among the Sabiny | Uganda | - *2 young women (15-24 years); *both were married; *both had some formal education; *both were Christian - *3 middle-aged women (25-35 years); all were married; *all had some formal education; *all were Christian - *3 older women (over 36 years); * all were married; *all had some formal education; *all were Christian | - Focus group discussions - In depth interviews | - Traditional childbirth and postnatal care at or near home - Formal childbirth and postnatal care in a health facility |
| Kyomuhendo 2003 | To enhance the understanding of why, when faced with complications of pregnancy or delivery, women still choose high risk options leading to severe morbidity and potentially death | Uganda | Women over 15 years of age; most were married | Focus group discussions | Traditional childbirth and postnatal care at or near home |
| Magoma et al. 2010 | To gain an understanding of the socio-cultural and health systems factors that influence women’s decisions to seek antenatal, skilled delivery and immediate post-partum care | Tanzania | 66 women seeking antenatal care, childbirth care and postnatal care at a health unit | - Focus group discussions - Key informant interviews | Traditional childbirth and postnatal care at or near home, especially for normal births |
| Mason et al. 2015 | To explore why some women access antenatal or delivery care in formal health facilities in the western Kenya context whilst many do not. | Kenya | - 18 adolescents (15-18 years) - 29 women of childbearing age (15-49 years) - 17 recently or currently pregnant women - 9 mothers of child born with an abnormality | Focus group discussions | Formal childbirth care in a health facility |
| Moyer et al. 2014 | To explore the impact of social factors on place of delivery, particularly on the impact of community and familial social structures and the role of cultural practices surrounding childbirth | Ghana | - 35 women with newborn infants - 81 grandmothers who had at least one grandchild within the past year of data collection | - In-depth interviews - Focus group discussions | - Traditional childbirth care at home - Formal childbirth care in a health facility |
| Myer & Harrison 2003 | To investigate factors affecting the utilisation of antenatal care services among pregnant women | South Africa | - 22 women (17-37 years) seeking antenatal care at a clinic; 14 women were married or in a committed relationship; majority of the women had formal education; 5 primigravidas - 7 women who had syphilis | Semi-structured interviews | Formal childbirth care in a health facility |
| Ndirima et al. 2018 | To understand women’s perceptions of the quality of non-clinical aspects of care that they consider important during childbirth | Rwanda | 20 women (18-43 years) who had delivered in the district hospital within 10 weeks prior to the start of the study; 10 women were primiparous (3 caesarean sections); 10 women were multiparous (3 caesarean sections) | In-depth interviews | Formal childbirth care in a health facility |
| Okafor et al. 2014 | To determine the preferred choice of maternity healthcare and determinants for pregnant and delivery services  among rural women | Nigeria | 25 women (20-42 years) who delivered a baby in the previous 2 years prior to the study; at least 13 women completed some formal education | Focus group discussions | - Traditional childbirth care in any domestic setting - Formal childbirth and postnatal care in a health facility |
| Osubor et al. 2006 | To assess maternal health services and health-seeking behavior | Nigeria | - Teenage girls (15-19 years); most were Christian - Women of childbearing age (20-49 years) and of parity of not more than 4 children; most women had some formal education; most women were Christian - Women in post-childbearing period (50 years and above); most women had some formal education; most women were Christian | Focus group discussions | - Traditional childbirth care in a traditional setting - Formal childbirth care in a health facility |
| Pfeiffer & Mwaipopo 2013 | To describe women’s health-seeking behavior and experiences regarding their use of antenatal and postnatal care as well as their rationale behind the choice of place and delivery  To learn about the use of traditional practices and resources applied by traditional birth attendants and how these might be linked to the biomedical health system | Tanzania | 100 women who delivered at a clinic or with the support of a TBA within 2 months prior to data collection; 49 women were married; 65 women had some formal education; 39 women were multiparous | - In-depth interviews - Focus group discussions | - Traditional childbirth care at or near home - Traditional childbirth care in a private and confidential environment - Formal childbirth care in a health facility |
| Seljeskog et al. 2006 | To identify the individual, community and health facility level factors influencing women’s choice of place of delivery | Malawi | 6 women of *childbearing age who had delivered recently; *all women were married; *All women had some formal education; 3 gave birth at home and 3 at a health facility | In depth interviews | - Traditional childbirth and postnatal care at or near home - Formal childbirth care in a health facility |
| Serizawa et al. 2014 | To explore cultural perceptions of and behaviors related to safe motherhood among Sudanese village women | Sudan | 6 women (16-40 years) of reproductive age who had given birth within 2-3 years prior to the study; all women were married; none completed any formal education; 2 of the younger women (16-30 years) were primiparous and multiparous; 4 of the older women (30-40 years) were multiparous | Semi structured interviews | Traditional childbirth and postnatal care at or near home |
| Shiferaw et al. 2013 | To understand why women might continue to prefer homebirths even when facility-based delivery are available at minimal cost | Ethiopia | 8 mothers (15-49 years); most women were married; most women were multiparous | Focus group discussions | - Traditional childbirth and postnatal care at or near home - Formal childbirth care in a health facility, especially for a complicated childbirth |
| Sialubanje et al. 2015 | To identify reasons motivating women to have homebirths and prefer the assistance of traditional birth attendants | Zambia | 100 women of reproductive age (15-45 years) who had given birth within 1 year prior to the study; 70 women were married; 93 women had some formal education;  50 were multiparous | Focus group discussions | - Traditional childbirth care at or near home - Formal childbirth care in a health facility |
| Sisay et al. 2014 | To explore beliefs and values surrounding neonatal mortality and stillbirth among several generations of rural Ethiopian women | Ethiopia | - 63 grandmothers who had given birth to at least 1 child, who in turn had given birth to at least 1 child; none had any formal education; majority of the women were Christian - 74 women who had any child under 5 years of age; all women were married; majority of the women were Christian - 70 younger women (adolescent girls over 15 years); none were married; all women had some formal education; Majority of the women were Christian | Focus group discussions | - Traditional childbirth care at home for normal childbirth - Formal childbirth care in a health facility, especially for a complicated childbirth |
| Thwala et al. 2012 | To explore and describe the values, beliefs, and experiences of rural Swazi women on childbearing in the postpartum period | Swaziland | 15 women (over 18 years) who had at least 1 child and whose last-born child was 2 years old or less; all women were married; most women had some formal education; *14 women were affiliated with tribal religions and 1 with Catholicism; all were multiparous | Unstructured interviews | - Traditional childbirth care at or near home - Formal childbirth care in a health facility |
| Wilunda et al. 2014 | To identify perceived barriers to utilization of institutional delivery care services in Moroto and Napak districts in Karamoka | Uganda | 459 women who had delivered in the past 5 years | Participatory rural appraisal | Traditional childbirth care at or near home |

* Additional data retrieved from authors of included studies.
