# Supplementary material for: Preferences for formal and traditional sources of childbirth and postnatal care among women in rural Africa: A systematic review
Source: PLoS One. 2019 Sep 25;14(9):e0222110. doi: 10.1371/journal.pone.0222110 (PMC6760778; doi:10.1371/journal.pone.0222110)
Supplement: S4 Appendix — Includes a table summarizing preferences and factors that influenced preferences. (DOCX) [file pone.0222110.s004.docx]

**S4 Appendix: Narrative Summary of Findings from Included Studies**

**S4 Table.** Narrative summary of findings from the included studies

| **Study** | **Preferences** | **Factors that Influenced Women’s Preferences** |
| --- | --- | --- |
| Adinew (60) | I. Majority preferred to give birth at or near home with traditional attendants | I. Some women feared clinical procedures such as vaginal examinations, which were perceived as uncomfortable and degrading. Women were most comfortable with the services provided by TBAs. In a traditional birth, women could give birth in familiar and comfortable positions, unlike in health facilities. Privacy is also a critical issue and one that is difficult to receive in health facilities due to the lack of private wards and cultural insensitivity. A traditional birth at or near home with TBAs enabled women to have greater control over their birthing environments, including privacy. In terms of reaching a facility, available transportation options were perceived to be too expensive and hard to find. Moreover, facility-based delivery services were too expensive, pushing patients to prefer the far more affordable TBA services. In addition, unlike health facilities, TBAs offered flexible repayment methods and time frames. Traditional childbirth care and TBAs were part of a long-time tradition for delivery and many want to continue engaging in such traditions. |
| Adinew (52) | I. Majority preferred to give birth at or near home with traditional attendants | I. Contributing to perceptions of quality, TBAs were said to care more about women’s feelings and treat them with more sympathy than health professionals, who were contrastingly perceived to be inconsiderate. Women also felt a strong bond with TBAs and had more trust in the TBAs due to their status in the community, prompting them to favor homebirths attended by TBAs over facility deliveries. Furthermore, a traditional homebirth favorably enables women to deliver in a familiar and convenient setting. TBAs also took cultural beliefs of their patients into consideration, while health facility staff members were less culturally sensitive. This included retention and burial rituals of the placenta, which was not possible in health facilities where they were disposed of by facility staff against the wishes of the new mothers. A traditional homebirth desirably enabled women to carry out the customary burial of their placenta around their home to avoid evil spirits and bad fortunes. |
| Ahmed et al (67) | I. Some women preferred to give birth at or near home. Preferences shifted to formal childbirth care when life-threatening complications arose  II. Most women preferred to receive assisted childbirth care in a health center facility | I. Some women preferred to give birth at home because it was the only type of childbirth they knew about. Others preferred a homebirth to adhere to social standards or generational traditions that mandate homebirths. Some people gave birth at home because they were afraid of what would transpire with a facility delivery, which many associated with treatment and complications management. Accordingly, the preferences of these women shifted when complications arose, their lives were threatened and there was no hope for a positive outcome without formal care.  II. Women preferred to deliver in a health facility (assisted birth) due to fears of complications. They believed a facility-based childbirth was the best choice for managing abnormal childbirths and ensuring a positive maternal outcome. |
| Allou (62) | I. All women preferred to give birth at or near home with a traditional birth attendant | I. Midwives had poor interpersonal relationships with service users. Midwives were said to shout at laboring women, treating them as if they did not know anything. TBAs were said to be more caring than trained midwives. This was because TBAs allowed women to choose their desired birthing position, whereas midwives chose the birthing position for pregnant women. Moreover, women who preferred to squat when giving birth were forced to lie down on a bed in the undesired lithotomy position. One pregnant woman explained how a TBA initially asked her to try the lithotomy position, but since the pregnant woman suffered in that first occasion, the TBA enabled her to give birth in the desired squat position in subsequent childbirths. A few women preferred TBAs because that was the only source of maternal care they were aware of that assists women during childbirth. These women believed that clinics were only necessary for when one became sick, such as a complicated birth. |
| Al-Mujtaba et al (36) | I. All women preferred to receive childbirth care in a hospital. The women expressed a preference for healthcare providers with accommodating attitudes over those with callous attitudes, and for expeditious services | I. Facility-based deliveries were favored for providing skilled care. For those who were HIV-positive, knowing their health status during pregnancy motivated them towards skilled childbirth. Providers who were perceived to be caring and compassionate throughout the pregnancy and childbirth were more accommodating than those who were perceived to be cruel, insensitive, and rude. As a result, they preferred to receive facility-based delivery services from providers with accommodating attitudes. Majority of the women had no gender or religious affiliation-based preferences, instead preferring quick and efficient facility-based services. These women were first and foremost concerned about receiving services expeditiously and their own health as well as that of their fetus. Locations and providers that provided quick health services were the ideal choice. |
| Bazzano et al (46) | I. Majority of the women preferred to give birth at or near home | I. Despite the high rate of use of facility-based antenatal services, the women reported that they had not received counseling or encouragement towards skilled childbirth, claiming that they also did not want to take up the nurses’ time by asking questions during their pregnancy. Even in traditional homebirths, they mentioned not wanting to ask for assistance, even from family members, because they were afraid of wasting someone’s time during labor. A fear of long labor also resonated to fears of being turned away from a facility if they arrived without being actively in labor. Preference for traditional homebirths was also based on fears of medical operations. A traditional homebirth was also preferred due to a lack of confidence in the hospital staffs’ attitudes and behaviors. They felt delivery at home without the attendance of a skilled provider was the ideal situation and was believed to embody a normal or simple childbirth. In addition, homebirths are highly valued, culturally. Traditional homebirths were also deemed to enhance their status within their family and community. Giving birth without skilled assistance was viewed as an accomplishment. In contrast, women who receive skilled assistance and formal childbirth care are seen as unfortunate and their status remains the same or decreases. Another reason was that laboring women who used facility-based deliveries were believed to be dishonest about the actual father of the baby. This label and associated stigma from facility deliveries encouraged traditional childbirths. Traditional childbirth outside of a facility also maintains secrecy of their birth, as it is believed to prevent enemies from practicing witchcraft against them. Traditional births at home were also less costly for families as facility deliveries incurred supply and transportation fees. Additionally, having an traditional childbirth does not disturb subsistence activities. |
| Bedford et al (44) | I. Some women preferred to give birth at or near home with the assistance of traditional birth attendants in an environment filled with their relatives and neighbors. This was particularly for ‘normal deliveries’ as facilities were preferred by these women when obstetric complications arose.  II. Some women preferred to receive childbirth care in health facilities in the presence of skilled providers, regardless of the perceived normalcy. | I. TBAs were preferred by some women because a ‘normal delivery’ was seen to be quick, easy and at home, surrounded by relatives and neighbors. TBAs were seen as a positive presence providing experience rather than skilled specialist assistance. Several women explained that it was their custom or habit to give birth at home, thereby precluding them from attending health facilities during childbirth. Also, the prohibition of relatives or neighbors from accompanying a woman in labor into the facility’s delivery unit, and associated feelings of loneliness during labor, further motivated women to deliver at home. Other reasons were that laboring mothers were able to move around freely at home, and to deliver in the kneeling position instead of the lying position that is instructed at health facilities, often with their legs in stirrups. The women felt that lying down with their legs up exposed their bodies to people they did not know. They desired the kneeling position as it was considered to be the most normal and dignified birthing position. Another key reason was the perception that a health facility is a place of illness and that ‘normal’ labor was concurrently a routine activity that did not require preparations, advanced planning, or a health facility. In consort, many expressed a sense of shame at having to attend a health facility rather than giving birth at home ‘normally.’ However, in situations where childbirth was abnormal, such as labor becoming protracted, their preference shifted to skilled care providers and for immediate medical intervention. Mothers who sought preventative health measures, such as contraception and vaccinations, also often opted for a traditional childbirth until labor was prolonged or complicated. Mothers felt that advice they had received from health professionals, such as preparing a clean sharp blade for cutting the umbilical cord, further encouraged them to deliver at home. However, some mothers who favored the traditional homebirths reasoned that they had never explicitly been instructed to seek skilled childbirth assistance at a health facility to begin with. For many, they also did not want to attend a facility due to the possibility of onwards referral and the lack of immediate treatment.  II. Those that preferred a skilled childbirth with the support of trained health professionals saw it to be beneficial for minimizing risks associated with home births. Additionally, younger mothers preferred to give birth at a health facility to avoid the pressure of delivering in front of relatives who might judge the progression of labor and their behavior. Lastly, mothers who received take-home educative material reported feeling better prepared for delivery and thereby encouraged to have a formal childbirth in a health facility in the future. |
| Caulfield (51) | I. Majority of the women preferred an traditional childbirth in a domestic setting in their own premises with traditional attendants | Many women were deterred from seeking facility-based services because of the low-quality services provided by professional birth attendants. Reports that health professionals left women alone during deliveries reduced the appeal of health facilities as women were customarily used to being held during delivery. Health professionals such as nurses also had a wide-spread reputation for having negative attitudes and being verbally and physically abusive to pastoralist women. Moreover, health professionals had a reputation of being careless of women’s desires and comfort. As a result, women had various reasons to adhere to their traditional birthing practices, which they viewed more positively. Some women had confidence in the abilities of TBAs and in the traditional herbs used by TBAs to help women who experienced problems during normal childbirth. For many, health facilities were merely places of illness that were only necessary for abnormal births with complications. TBAs were described as highly valued and revered community members. Since TBAs were part of the community, women were also more familiar and comfortable and trusting of them as attendants to childbirth. A traditional birth was favored by some women because it was more comfortable than delivering in health facilities. In a traditional birth setting, women received physical and social support from loved ones. Moreover, women also preferred a homebirth because facility-based deliveries required them to be naked, which was considered to be dishonorable. During homebirths, TBAs took women’s privacy into consideration by providing cover to women with a blanket or sheet so that their bodies are not exposed. A traditional birth also enabled women to control the surrounding environment and to conceal non-circumcision from others. In terms of accessibility, TBAs were readily available and far more accessible than facility attendants. Health facilities were also not favored because of restrictions on family support in labor wards. They did not accommodate pregnant women’s requirements of support and consolation during deliveries, either by providing the support themselves or enabling trusted support casts into the labor ward. On the other hand, a traditional homebirth enabled women to receive the support and consolation that they desired, including hand holding. Women suggested that having family members help with the delivery ensured that a baby is delivered easily and that this was why they wanted a traditional birth. With considerations of social status, women wanted to deliver at home because a professionally assisted delivery was seen as weak, while a traditional delivery was seen as courageous. A home delivery through self-care was particularly labelled as a standard practice and a brave birth, leading some women to desire this feat. Some women reasoned that their preference for homebirths was related to its customary status in that it has always been the norm and the standard way of delivering in their culture. Tradition was very important to these women and in the popularity of traditional births. Specific cultural practices and beliefs also influenced women’s preferences for homebirths. Some women said that a customary announcement is made when a baby is born, which cannot be done at a hospital. Others identified superstitious beliefs such as ensuring blood loss during delivery is kept within the homestead to protect against bewitchment. Accordingly, TBAs were preferred because they respected and took consideration of cultural practices and birth rituals. There was a sign of potential change in the future as younger women were said to prefer hospitals more than older women. |
| Chea et al (61) | I. Most women preferred facility-based childbirth care in a hospital facility | I. Health facilities had the necessary equipment and supplies required to conduct deliveries. Also, doctors and other professional attendants in hospitals were believed to have the skills to assist a normal childbirth and the capacity to handle a complicated childbirth. Some women also wanted to deliver in a facility because they believed it helped prevent transmission of HIV infection from mother to child. In contrast, traditional attendants at home were unskilled and thereby incapable of managing childbirths, especially when complications arose or there were concerns about transmission of HIV from mother to child. Facility attendants also favorably educated and advised women about various maternal health and child health matters, such as breastfeeding practices. Education and advice were not available from traditional attendants. Some women wanted a facility delivery because they experienced labor mismanagement, harassment, stigma and discrimination at home with traditional attendants. This stigma and discrimination was said to be associated with their HIV status. The reception at the hospital was also credited for receiving patients well on arrival. |
| Cofie et al (50) | I. Some women preferred to give birth at home as their first line of care. They preferred to receive childbirth care in a facility as a last resort when complications arose.    II. Some women preferred to receive childbirth and postnatal care in a facility, as their first line of care. | I. Successful previous experiences with traditional births encouraged many to deliver at home. Looking back on those experiences, they associated homebirths with easy labor and delivery. Negligent and denigrating skilled birth attendants who were staffing certain health centers deterred women from seeking skilled assistance. Overall, childbirth care in a facility was only preferred as a last resort during the onset of complications, as they believed facilities and trained providers were better equipped to manage their complications. Culturally, many women preferred a traditional childbirth because homebirths were the village norm spanning generations. Home births were also cheaper and more convenient than facilities, which required travel expenses and long distances to cover. Some preferred home deliveries because poor roads and modes of transportation, such as motorcycles, were deemed unreliable and detrimental to women’s health.  II. Some women preferred health facility births and facility-based postnatal care thereafter due to their awareness of health risks associated with childbirth. They expressed concerns about risks associated with homebirths and the need for skilled care for birth complications. They believed facilities helped them avoid the consequences of potential obstetric complications during childbirth and the postpartum period. Preference for a skilled childbirth in a health facility for some women was based on whether their previous pregnancies in a facility resulted in births. If their previous children were born there, they preferred to give birth in a facility again. They were also encouraged to use health facilities that were staffed with helpful and caring providers, especially midwives. Lastly, a reason for preference of facility births included the perceived high prevalence of, or a shift in norms towards, skilled childbirth in a facility. |
| Dahlberg et al (31) | I. A minority preferred give birth at home with traditional attendants  II. Nearly all mothers preferred to receive childbirth care in a health facility. They want health professionals to provide close-care. Some women specifically preferred Muslim facility-based attendants to facilitate their deliveries | I. A minority preferred a traditional childbirth due to the perceived negligence and insensitivity of health care providers, and the abusive environment of a health facility. Giving birth in a health facility also came with other challenges, such as how to obtain appropriate food (e.g. halal meals), particularly when the facility was too far from home or from relatives and friends. It was reported that some healthcare providers did not understand the importance of halal meals to Muslim women, thereby encouraging women to seek traditional care outside of a facility. Traditional births were also preferred for uncomplicated childbirth, with the preference shifting to skilled childbirth care for complicated childbirth. Although most of the female relatives had experiences of both homebirths and facility-based deliveries, they described traditional births at home as the best option. They said that it was acceptable to start at home, and that only in the case of complications or if the mothers were HIV positive would they support facility delivery**.**  There was also a perception among the mothers and the healthcare staff that payment to TBAs could be delayed or entirely avoided. Additionally, the sociocultural impact of the TBAs and the possibility of surveillance of the TBAs’ work were some of the reasons described by women, including older female relatives, for favoring childbirth care at home.  II. Mothers who were HIV negative preferred a skilled facility-based delivery out of fears of infection, while mothers who were HIV positive preferred a facility delivery due to a fear of infecting the child or the traditional birth attendant. Mothers who were HIV positive sought facility-based deliveries. TBAs were said to lack modern delivery skills and timely referral capacities, creating obstacles for safe deliveries. They also believed that facility-based attendants would be better able to solve acute complications. Though preferring modern childbirth care, some women indicated their admiration of how TBAs bathed them in warm water and provided close care and comfort, whereas the facility-based attendants bathed them with cold water. They desired this type of care from health professionals. The perceived incompetence and insensitivity of some facility providers to Muslim women's maternity care needs and religious and cultural practices was a key deterrent. Even when healthcare providers were aware of the religious practices and maternity care requirements of Muslim women, it was reported that they did not consistently take their needs and concerns into account, with some providers becoming irritated with the women for emphasizing their religious needs. As a result, some women preferred skilled childbirth in a facility with the assistance of Muslim health professionals, feeling they would be better able to provide competent and religiously sensitive evidence-based care that addresses their maternity needs. |
| De Allegri et al (55) | I. Some women preferred to give birth at home in their own village.  II. Women preferred to receive childbirth and early postnatal care in a health facility. | I. Some women desired traditional homebirths because of the traditional belief that delivery should occur in one’s own village. Facility-based deliveries were systematically avoided, not just because of fears of an institutional delivery, but also out of a wish to remain in one’s own village during labor and delivery. Preference for a traditional childbirth over skilled childbirth was also related to a recent case in the village of maternal death having occurred at the facility, leaving village members to speculate on the causes of such death and develop skepticism towards facilities.  II. Women understood and valued the benefits of delivering in a health care facility. They believed facilities can deal better with health complications, such as removing blood from the belly. A homebirth was associated with a slow recovery, weakness and lethargy, which thereafter would negatively impact their ability to complete tasks. |
| Dodzo & Mhloyi (45) | I. Majority preferred to give birth in traditional community centers with community-based traditional attendants  II. Some women preferred to give birth and have traditional postnatal care in their own home with or without assistance.  III. Most women preferred to have traditional postnatal care in traditional community centers with community-based traditional attendants. | I. Community deliveries under the guidance of traditional community practitioners were preferred due to lower transportation costs**,** which included regular transportation fares to the facility and additional emergency referral transportation fares. With traditional community deliveries, user fees are minimal or non-existent, as community birth attendants, such as TBAs, do not require administration pay for childbirth services not directly linked to the maternal services offered. On the other hand, clinics and hospitals charge for consultation and professional fees, as well as extra costs that come from complicated childbirth. Some women also misunderstood booking and the booking fee. As a result, real and perceived costs were key reasons why women preferred to receive traditional maternal services in the community. Community attendants were also preferred because they offered more flexible payment options than facility-based providers. Unlike health professionals, they do not require up-front payment due to flexibility in repayment time-frames; they were additionally willing to accept livestock, poultry, grain, labor or other social favors as payment, long after the service is provided. Community-based attendants also offer negotiated payment terms based on service results, whereby the women pay them after confirming the desired results. The women found this to be very accommodating and patronized how emergencies are attended to first before discussions about payment, making them feel that community attendants cared more about their clients’ welfare than payment. Another reason was the reduced social and opportunity costs from staying near home in the community, since attending health facilities meant leaving behind multiple household chores and responsibilities that are crucial to the welfare of the family**.** Staying at home kept household chores from doubling or tripling, as was believed to happen when hospitalized. Moreover, delivering in a health facility was perceived to deny the family and the woman the social, emotional and psychosocial benefits of a family gathering during childbirth and following childbirth with the neonate. Women were also concerned about leaving their husbands alone for prolonged periods as they believed it could result in unfaithfulness, HIV infection, family malfunction and divorce. Women liked how community attendants allow them to go back and check on the welfare of their families. In contrast, facilities have restrictions and social curfews. Women claimed health facilities do not provide culturally and religiously-sensitive services, which were important for preventing misfortune on their babies. The community was said to be the best venue for observing cultural and religious beliefs during the provision of maternal health services. Previous positive community deliveries also motivated women to repeatedly opt for community maternal care services. Another reason why women prefer community deliveries comes from their beliefs of the origin of maternal complications and hospital care. Complications are not expected because of the belief that when pregnancy is desired by the woman, there will be no misfortunes during delivery. As a result, women are convinced that they can deliver in their traditional setting without skilled assistance. They believed complicated births are cursed, of spiritual origin around the delivery period, and that religious intervention from a spiritual or traditional healer is the best solution. Moreover, they perceived health facilities as treatment centers instead of locations for management of pregnancy and childbirth. As pregnancy is not conceived as a disease, they further saw no need to attend a health facility.  II. Women who felt they could not abandon their household chores to obtain services in a traditional community center or a facility preferred to receive childbirth and postnatal care at home. Traditional centers and facilities were perceived to be too far away to enable them to maintain their household responsibilities. Receiving maternal health services in their home saved them significant financial and opportunity costs. It also enabled them to have complete control over their households and brought them a peace of mind throughout the maternal periods.  III. Although the desire for a healthy baby is a motivation for seeking postnatal care at health facilities, they did not trust the health care system with the unseen baby. They trusted traditional or spiritual attendants with their unseen baby, leading them to prefer postnatal care from a traditional community center. Women felt they could not abandon their household chores to obtain services in a traditional community center or a facility with the added responsibility of looking after a newborn. Some women also did not want to leave home due to concerns of unfaithfulness by their husbands, who were described as ineffective in household and family management in the absence of the wife. |
| Engmann et al (49) | I. All women expressed a preference for receiving childbirth care in a healthcare facility from health professionals. | I. A key reason for their preference was the nascent norm regarding childbirth in the community. Whereas the traditional homebirths used to be the norm, high mortality rates and increased awareness of the dangers of pregnancy and delivery encouraged women to seek care in health facilities for a skilled childbirth. Health facilities were seen as the only settings where ‘hidden illnesses’ or complications were detectable. They also believed in the ability of skilled birth attendants over TBAs to mitigate such dangers. Some women also preferred skilled childbirth because influential community members, such as members from church, promoted facility-based deliveries. Even women who experienced complications and/or poor pregnancy outcomes after delivering at a facility favored and endorsed facility-based childbirth care. Primigravidas expressed that they favored health facility delivery because they could not receive skilled assistance out of the health facility. |
| Ganle (47) | I. Many Muslim women preferred to give birth at or near home with traditional attendants (TBAs, relatives).  II. Some women preferred to receive care in a health facility from health professionals. They prefer these providers to be familiar and understand their religious beliefs, practices and maternity needs. This included a preference for Muslim attendants and female attendants over male attendants for antenatal and childbirth care. Others expressed a preference of receiving maternal health services from skilled, loving and empathetic providers in a health facility. | I. Many women preferred traditional care outside of a facility and in a domestic setting in order to fulfill religious beliefs. They preferred to stay at home due to the significance of maintaining the sanctity of the female body in Islam, which they believed required them to keep their physical body away from the public eye. This was especially an issue when male nurses, midwives or doctors, who they did not have an intimate relationship with, were able to see their naked body. Muslim women were deterred from labor wards due to related concerns of maintaining privacy. They felt the facilities do not prevent other people from seeing the laboring mothers exposed. They preferred traditional childbirth care with the support of TBAs and other family members because their perineal area was covered with a piece of cloth and the room usually only had women, who tended to avoid looking at the laboring mother’s genitalia while delivering the baby. The lack of a quiet place for prayer was another factor that was repellent to many women.  II. Pregnant women were said to need love and empathy from health care providers and relatives to have a safe childbirth. With fears of dying while giving birth, a few women felt their maternity needs would be best met with professional assistance in a health facility. Facilities that respect women’s religious maternity needs and match them up with the compatible attendants enabled the women to receive skilled antenatal and childbirth care. Muslim attendants were preferred because that enabled them to be religiously and culturally matched up with a provider that they perceived understood their plight and shared their faith. Some also wanted to use facilities to enable them to fulfill a religious obligation to circumcise their male infants. Female attendants best enabled the women to sustain the sanctity of their bodies. In consort, this enabled them to follow perceived religious rules and duties in Islam. Successful childbirth, which was believed to be more likely with skilled assistance, brought them honor and guaranteed a place in their polygynous environment. |
| Ibrhim (63) | I. Majority of the women preferred to give birth in a domestic setting | I. Lack of equipment, supplies, and drugs necessary for maternal care in health facilities contributed to women’s preference for home deliveries.  In addition, despite the advertisement of facility delivery services being free of charges, women still had to spend to get medicine and other required supplies due to the shortages. Some women wanted to avoid health facilities due to health professionals that lacked proper skills and confidence to assist a delivery. Women also complained about the rudeness of some health professionals. Some women reasoned that a facility-based childbirth under the guidance of a skilled attendant does not necessarily mean that they would avoid maternal deaths and other poor outcomes, as in traditional homebirths. Majority of the women lacked information and had no awareness or knowledge about the availability and benefits of delivery facilities. Women were also dissuaded from using a health facility due to the lack of privacy, since they had to expose their private parts to various staff. At home, they were favorably able to control the degree of privacy during labor and delivery. Majority perceived childbirth as a natural process that should take place at home following local customs and traditions. Accordingly, a health facility was only perceived to be necessary as a last resort for when complications arose. TBAs were believed to have the capacity to advise a family to take a laboring mother to a health facility when complications arise. Most women indicated that distance to health facilities made homebirths the easier option. The remote nature and dry conditions also made it difficult to walk to facilities. For some, health facilities were too far away from where they lived. The pastoralist way of life involves living in scattered drylands with no permanent residence, constantly moving from place to place to find grazing land and water daily. This way of life and its challenges affect maternity in that health facilities are not organized and accessible enough to service such mobile populations. Lack of transportation options was another reason women wanted to deliver at home. Poor previous experiences during labor that involved long waiting times and no transportation options to go back home also dissuaded some women from seeking facility-based care. Additionally, the cost of transportation expenses, food and drugs were financial constraints that motivated women to stay home for childbirth. Drugs were not always available in health facilities and needed to be purchased from pharmacies. Additionally, during emergencies, many indicated that emergency ambulance services were not readily available and accessible. |
| Igboanugo & Martin (40) | I. Some preferred to give birth at home with native doctors or in spiritual healing homes with spiritual attendants.    II. Majority preferred to receive childbirth care in private health facilities over public health facilities.  III. Some preferred to receive childbirth care from skilled professionals in a government facility over native doctors and spiritual healing homes. | I. The skepticism of modern medicine and perceived inhospitable attitudes of health facility staff towards pregnant women motivated women to avoid health facilities; instead, they favored and sought traditional care from spiritualists and native doctors who provided traditional childbirth services. They had complete faith in the spiritual healers and native doctors. Cheaper services were another reason they preferred traditional care.  II. Private modern care was preferred because it was perceived to be more affordable and to enable women control of their own choices during labor and delivery. They felt the private facilities reflected the desires and opinions of the women in the services provided. For others, private hospitals were cleaner as they were paying for it, while the perceived cheaper public hospitals were not clean. Also, the long queues for public facilities encouraged them to attend private facilities for service that is faster and of higher quality.  III. Some women did not trust healing homes, but instead preferred government health facilities because they did not believe healing homes had the appropriate equipment to deal with pregnancy and delivery. These women doubted the competency of healers as they lack medical professional training and instead rely on superstition and their experience. Health professionals on the other hand were trained and thereby believed to be competent in guiding their childbirth. |
| Kea et al. (64) | I. Majority preferred to give birth childbirth in a domestic setting | I. Despite the availability of health professionals and health extension workers nearby, traditional childbirth care and attendants were the preferred choice over formal care due to familiarity, comfort and privacy. Some women did not want to visit unfamiliar settings where they would have to expose their bodies to strangers, such as health professionals. Several indicated that they would prefer the presence of their husband and relatives during their delivery than health professionals. Moreover, a homebirth was more appealing as it gave them privacy from strangers. Women also held concerns about being forced to lie down and stretch their legs in a delivery bed by facility staff over the preferred customary squat position that is taken during traditional homebirths. The lithotomy position was believed to both expose them and make them vulnerable to illnesses. |
| King et al (41) | I. Some women preferred to give birth at or near home with the support of traditional attendants  II. Some women preferred to receive childbirth care in a hospital facility with accepting and considerate health professionals. | I. Most reasons were largely based on health facility deterrents. Their perception of poor quality facility-based care, such as the provision of repeated examinations by many different staff, angered women and invigorated them to seek a traditional birth at home. The perceived negative and offensive behavior of skilled providers associated facility care services with unprofessionalism and culturally unsafe practices. In contrast, TBAs were respected, admired, comforting and seen as relatives by some women. Nurses were not trusted and were believed to expose their clients. TBAs were trusted to protect and maintain the privacy, integrity, security and safety of the women. Infact, TBAs were respected to the degree that they broke gender barriers and were part of the community decision makers. Many women preferred to deliver quietly at home with a female TBA because they are shy and secretive about pregnancy and birthing. TBAs were perceived to provide more affordable, safe and culturally appropriate care than skilled attendants. They also had a cultural preference for traditional care from TBAs as TBAs had always been used in their community by their predecessors.  II. Hospitals were credited for being clean and well-equipped. Women also supported health-care providers for their attitudes and encouraging advice for further attendance. Providers who accepted women’s desires during labor and delivery encouraged the women to attend the facility in the future. At times, well-equipped facilities, in terms of medical supplies and accommodating personnel, were sometimes beyond the women’s community, forcing some women to prefer and seek better-equipped facilities in another community. |
| Kumbani et al (56) | I. Women preferred to receive childbirth in a health facility with the close support of a skilled, respectful and reserved provider. | I. Women preferred a facility-based skilled childbirth because traditional care with a TBA was not considered adequate. TBAs were believed to lack knowledge about maternal problems and the competence to manage complications. Also, some women were not happy with the tardiness of TBAs and being left along during labor when they were in pain in previous experiences. In contrast, they felt they received adequate care at a health facility, with access to medicine or workers with the capacity to refer them to higher levels of care. Participants believed that readily available attendants would help them avoid self-deliveries and that respectful providers would encourage them to attend facilities for maternal services. |
| Kwagala (65) | I. Most women preferred to give birth in a domestic setting with traditional attendants  II. Most women preferred to have traditional postnatal care at or near home  III. Some women preferred to receive facility-based childbirth care and postnatal care | I. TBAs were praised for providing comprehensive and consistent childbirth care while also considering cherished cultural practices. Women had excellent interpersonal relationships with TBAs, who were characterized as polite and comforting care-takers. They provided warm water for bathing, meals, clothing, and they held hands and gave women strength to keep pushing. Sabiny women also preferred traditional births because they traditionally squat, kneel or sit to facilitate delivery, whereas health facilities would compel them to lie down in the supine position. This position during delivery was perceived as an unsafe position that could kill the baby. The preference for traditional birthing positions is also associated with the desire of circumcised women to limit the exposure of their bodies and maintain privacy. They were also favored because they accepted women regardless of their financial prowess and hygiene. Most women also preferred traditional births because they do not want to be seen by non-Sabiny health staff members that are strangers to the Sabiny culture. Women also wanted to stay at home in order to perform postnatal rituals when the baby is born. Many wanted to be aided by those who they trusted, who tended to be traditional care-takers. This trust is illustrated when advice given by health professionals is often subject to a TBAs approval. In addition to the physical, emotional, and social support, a traditional homebirth favorably featured additional social support provided by family members and other community members during delivery. In contrast, health facilities did not authorize such family and community members in labor wards to provide support and advice. Unassisted homebirths came with high levels of social reverence. They were seen as endurance tests and as markers of a real woman. In contrast, a facility delivery was seen as a sign of weakness because the women had to rely on drugs and equipment to easy the process of childbirth. It relinquished a woman’s desired identity as a strong woman. There was a strong cultural preference for births in a domestic traditional setting, including women’s homes and the homes of TBAs. The application of herbs, the mobility of the woman in labor to facilitate the descent of the baby, and other cultural practices are all more accepted and achievable at home than in health facilities. There is a Sabiny ideal that is based on a story that the mother of the 10 NiloHamitic groups, including the Sabiny, who migrated from Ethiopia had non-institutional and unassisted births that the daughters should emulate. In consort, women preferred homebirths because as perceived daughters of that mother, they want to emulate her maternity choices. The placenta is seen as a baby in another form that requires proper disposal in order to keep the newborn from becoming ill. In a traditional childbirth, women can favorably bury the placenta in a banana plantation or outside their house, or throw it into a pit latrine facing upwards by a trusted community member.  II. TBAs were praised for providing comprehensive and consistent postnatal care, while also considering cherished cultural practices. Women preferred a traditional PNC in the early period due to key postnatal cultural practices that must be performed. This included clamping the baby’s umbilical cord and applying charcoal powder and herbal extracts to the cord stump. Boiled water, sugar and salt are then given to babies to cleanse their stomachs and ease digestion. They are also given herbal mixtures to boost their immunity. Moreover, TBAs provide ritualistic care to the baby and mother, which generate a sense of belonging to the community. The placenta of a baby born in the breech position, which is perceived as a curse, must be pinned on a stick and hanged in prayer on the roof of a home. This action is associated with forgiveness from the ancestors and the blowing away of the curse by the wind. Traditionally, the mother and the newborn must remain in seclusion at home during postpartum for at least a week after delivery. The baby is kept from going out of the home compound in order to avoid people with the ‘evil eye’ and resultant disease afflictions. It also gives the mother time to recover from delivery in the comfort of her home. This tradition can go up to three months if a mother bares twins, which would include the in-house seclusion of the mother, the babies and the placentas.  III. Some women preferred to deliver in a formal health setting with mature, female health attendants from their own culture or at least a facility attendant that was familiar with the Sabiny culture and willing to follow-up patients in the community. Many women also preferred a facility delivery when critical complications arose. Some women preferred institutional postnatal care when critical illnesses and complications arose in puerperium. |
| Kyomuhendo (43) | I. Majority preferred to give birth and to have traditional postnatal care traditional at or near home with traditional attendants | I.  Health professionals, especially nurse-midwives, were said to be disrespectful, poorly trained, expecting bribery, and deliberately negligent to certain patients. Women also complained about young health professionals telling them what to do in the facility, as it made them feel passive and foolish. They also viewed that skilled childbirth at a facility was an anti-climax, while traditional options were more climactic. They expressed government health services as places where impersonal service providers focused on efficiency, hygiene, orderliness and the suppression of mother’s emotions, which the women believed to interfere with their normal birthing process. Unlike in facilities, when pains came, women could respond how they wished, without restraints or forced hurrying. This is traditionally part of a woman’s upbringing, and women giving birth consider the free expression of pain as an integral component of childbearing. Having to suppress this not only undermined the meaning and value women attached to the experience but also made them feel that the sensations taking place within their bodies were being disparaged. Another reason was the positions that the facility providers compelled them to adopt for delivery at hospitals, whereas homebirths enabled them to deliver in their preferred kneeling positions. A key factor was that health workers were often seen as strangers or outsiders and not recognized as part of the local birth culture. TBAs, grandmothers and traditional healers were instead considered the main providers of the local birth culture. TBAs and relatives were known or seen as fellow community members, with their maternity services being familiar and acceptable in the community. The delivery of formal maternal care was not perceived as culturally appropriate, and the perception of maternity care providers at government health facilities was negative. Women were also incentivized to deliver at home because traditional childbirths, especially when complicated, were deemed courageous and earned the respect of their community, thereby enhancing their social status. |
| Magoma et al (32) | I. Many preferred to give birth and have traditional postnatal care at or near home with traditional attendants, particularly for births without complications.  II. From those who intended to seek childbirth in a health facility, majority preferred district hospitals over local dispensaries. | I. A main reason was women’s perceptions of the ‘naturalness’ and safety of home deliveries. For ‘normal pregnancies’ as defined during ANC visits, women sought traditional home care believing it would still result in successful deliveries. Mothers and grandmothers also had traditional homebirths without skilled assistance, which encouraged many women to do the same. They believed that health facility deliveries were only beneficial for women with complications, as a last resort after developing serious complications. For some women who preferred unassisted homebirths, transportation to health units for delivery or emergency obstetrical care was considered unreliable and/or unaffordable. TBAs and relatives were viewed by the women as affordable, convenient, and able to meet their service expectations. These expectations included continual support and advice throughout the continuum of care, body massaging throughout labor and delivery, and knowledge of a variety of delivery positions**.** In contrast, women did not believe health facilities can provide these services, where the environment and languages are foreign to them, and the few available providers are over-tasked. Most women were concerned about, and deterred by, the prospect of certain routines and life-saving procedures conducted at health facilities during labor, delivery and the immediate postpartum period. Women feared undergoing caesarean sections with no explanations in advance from providers in a facility. They also feared episiotomies and repairs of genital tears sustained during delivery from seeking skilled childbirth care. Genital tears were viewed as inevitable complications of childbirth that did not require medical intervention. Some perceived vaginal examinations at facilities, especially when done by men, to be dehumanizing, painful and harmful for the baby, potentially causing labor retraction. Conversely, TBAs were preferred because their vaginal examinations were perceived to be gentle and only done when the baby’s head is crowning. Insufficient dialogue on the importance of skilled childbirth care was also a reason why traditional care was preferred. They felt the providers did not talk about the importance of health unit deliveries, particularly during ANC visits. This was in contrast to TBAs, who often told women to go to health units when complications arose. In terms of social roles, the need to resume household responsibilities soon after delivery was key reasons women preferred a traditional homebirth and traditional early puerperium. A minority of women had a traditional belief that regardless of where they delivered, complications and maternal deaths would occur on those being punished for past transgressions. Therefore, they did not see the point of attending a health facility even during complicated childbirth as only ‘God’ can protect them from maternal death.  II. Previous experiences in nearby dispensaries, which provide some primary care services as provided by health centers, convinced women that they were not suitable places to seek childbirth care. In contrast to dispensaries, they trusted district hospitals to best manage complications during the intrapartum period. |
| Mason et al (34) | I. A few women preferred to give birth at or near home with traditional attendants  II. Majority of the women preferred to receive childbirth care and postnatal care in a health facility. | I. If a pregnant woman was healthy, they preferred to undergo a traditional childbirth as it was deemed unnecessary to seek professional assistance in a hospital. It was only deemed necessary to seek a facility-based childbirth if a pregnant woman was experiencing complications during the childbirth. Others preferred to give birth at home or at an older relative’s home with a TBA for various reasons, including high costs, distance to health facilities, and lack of transport. Traditional care at or near home was cheaper, close to their own residence, and did not require much transport, with many women otherwise needing to travel up to 5 km to access a hospital.  II. Overwhelming response about the preferred setting for delivery was the hospital. This preference was primarily due to an understanding that if complications occurred either during the delivery or in the postpartum period, the hospital was the only setting where they could be managed. Excessive bleeding, retained placenta, and having a large or badly positioned child were specifically referred as complications best handled with facility-based care and professional childbirth attendance. TBAs on the other hand were not believed to have the capacity and ability to manage complications (TBAs cannot handle complications). One woman preferred a facility-based childbirth because she was HIV positive. Any obstetric issues associated with HIV would be best managed by hospital attendants |
| Moyer et al (48) | I. Some women preferred to give birth at home  II. Most women preferred to receive childbirth care in a facility from trained professionals. | I. The preference for traditional childbirth care was based on traditional beliefs and associated fears. Skilled childbirth in a facility was believed to be a taboo and to bring negative consequences, including maternal or infant death**.** This was typically expressed in families practicing a traditional religion in which soothsayers were consulted before women could go to a facility. Families feared repercussions from the ancestors if they went against the soothsayer’s advice for childbirth.  II. The norm regarding the place of delivery has changed from homebirths to skilled facility-based childbirth. Respondents cited safety and the prevention of death as key reasons for why they wanted a skilled childbirth in a health facility. They also accredited their choice to the counsel received from community health workers and from attendants during ANC visits. |
| Myer & Harrison (39) | I. Majority preferred to receive childbirth care in a facility. | I. Most women believed that childbirth was a high-risk event linked to obstetric complications, such as excessive bleeding during labor and delivery. They perceived labor and delivery to require biomedical attention. Therefore, they preferred facility-based deliveries, believing that modern care and skilled assistance best provided positive outcomes for the mother and the baby. |
| Ndirima et al (33) | I. Most preferred to receive childbirth care in a facility from community health workers (e.g. midwives) to assist their childbirth. They further preferred wards with professional community health workers. Some women specifically preferred male providers, while others preferred female providers to assist their childbirth. Most women preferred to receive facility-based childbirth care in a secluded and private location. Some women preferred to be accompanied by their mothers-in-law over their husbands, while others preferred the opposite. | I. They were confident in the training and competence of hospital-based health professionals for detecting and managing complications. However, they did have confidence in the competence of interns. Some women also wanted hospital deliveries because previous deliveries had been caesarean sections, while other women wanted hospital-based care after previous delivery failures at home with TBAs. Some preferred female health attendants for privacy related reasons. Such women felt discomfort with some procedures performed by male attendants or multiple individuals. Female attendants helped them feel that their privacy was protected. Additionally, they felt they could better relate to female attendants for knowing exactly what they were going through. On the other hand, some women preferred male attendants because they believed male attendants knew more than female attendants. They also believed that men were emotionally stronger and empathetic to help in complicated maternal situations. Several women were worried about giving birth in crowded labor wards that often lacked medical and human resources. They were embarrassed to give birth in an open place where their private parts were exposed to strangers, making them feel their privacy was not being protected. As a result, they wanted to deliver in a hospital, as long as it respected the need for privacy during childbirth. Some women, especially those who were multiparous, expressed a preference for assistance from their mothers-in-law over their husbands because mothers-in-law could nurse them better and these women considered delivery to be a feminine act. The women also feared their husbands would become less sexually attracted to them after observing the childbirth. Others however wanted their husbands to be by their side during delivery for emotional support, physical support, and decision-making and logistical support. They felt more secure and relaxed, which was an important condition for the appeal of a facility delivery. |
| Okafor et al (35) | I. Some women preferred to give birth in a traditional setting with traditional attendants  II. Some women preferred to receive childbirth care and early postnatal care in a facility setting from trained health professionals. Majority of these women preferred general hospitals or government facilities for childbirth care. | I. Childbirth care service preferences were situationally based on prevailing circumstances for the majority of participants. A key reason for the preference of traditional childbirth care from TBAs is that TBAs are perceived to possess special skills, including massaging, identifying the position of the baby and correcting the position if necessary. TBAs were critically believed to help them avoid complications during childbirth. They were said to prevent miscarriage and some diseases which affect children and pregnant women in the intrapartum period. Also, procedures of episiotomy and caesarian section were perceived as complications, and delivery with TBAs was believed to help them avoid these perceived complications. For some women, these complications were believed to only be cured or managed by the TBA and not by skilled providers or tests in the hospital. From a traditional perspective, the women identified a special concoction (‘aseje’) as a key attraction for seeking care from TBAs. This concoction was believed to prevent development of any complications during labor to keep pregnant women healthy. Another concoction (‘agbo’) given by TBAs was also believed to develop a small baby and allow an easy delivery. In contrast to the herbal mixtures provided by TBAs, the injections and tablets provided by formal providers were disliked by most women, discouraging them from formal care. They also preferred to give birth with assistance from TBAs because it is cheaper. Traditional beliefs and cheaper services encouraged them to seek traditional care.  II. Some women preferred skilled childbirth care due to the provision of medical checks and vaccinations and the management of labor and delivery complications. They also received ultra sound scans and tests. Women further preferred skilled providers for labor because with an injection they can deliver the baby within a short time, while immunization for their babies in early puerperium was also deemed significant. Some women prefer the hospital for checking the position of the baby instead of TBAs as they believed the formal providers were more equipped and had the capacity to do so. Many especially wanted to use the hospital in situations where they experienced complications, such as prolonged labor. Some women preferred government hospitals because they were cheaper than other formal services. Several women simply sought to receive care from either a facility or a TBA based on the most convenient date available. |
| Osubor et al (42) | I. Some women wanted to give birth in a traditional setting with traditional attendants  II. Some women preferred to receive childbirth care in private facility-based maternity centers from trained attendants over government-owned clinics. | I. Their preference of traditional and spiritual care was influenced by the perceived quality of care**,** largely based on perceptions of the service providers’ behavior. TBAs were considered to be more affectionate and caring compared to the staff of the government facility. Essentially, they had far better interpersonal relationships with TBAs. A fear of facility-based operations was cited by those who particularly sought TBAs rather than trained health professionals. Another reason was the waiting time, which was considered to be longest in the government facility. Some women also felt more comfortable having their birth supervised by a TBA. In addition, TBAs were favored because they allowed the women to give birth in their desired and most comfortable positions. Abnormal and unnatural health problems, which were seen as traditional or spiritual health problems, were perceived to be due to witchcraft, and such were believed to be best handled by traditional healers and spiritualists. Preferences shifted for normal and natural health problems, which were believed to be best handled by facility-based care.  II. Skilled private care was believed to be of higher quality, largely based on perceptions of the service providers’ behavior. Private facility midwives were considered to be more affectionate and caring compared to the staff of the government facility. The waiting time was also desirably shorter in private facilities and longest in the government facilities. Women felt more comfortable in private settings and with private midwives. Normal and natural health problems, seen as physical illnesses, were believed to be best detected and treated by formal medicine, preferably in a private facility. |
| Pfeiffer & Mwaipopo (54) | I. Some women, most of whom were younger women, preferred to give birth in their homes or traditional maternity centers with traditional attendants.  II. Some women preferred give birth in a private and confidential environment with the assistance of someone from within their community.  III. Some women preferred to receive childbirth care in a facility with trained birth attendants. | I. The main factor for delivering at home or in a traditional maternity center was convenience**,** as their homes or the operating centers of TBAs were located closer than a health facility. Additionally, the low fatality rate from home deliveries encouraged younger women to believe in the safety of delivering at home without skilled assistance.  II. A private environment was preferred by those who wanted to hide their pregnancy for cultural reasons, such as a custom of starting labor at home. Health facilities were considered to be public spaces that lack privacy and suitability. These women shunned health facilities in favor of community deliveries for confidentiality. Being assisted with a member from the community allowed them to receive care from someone they know and trust. This member was often a community based TBA.  III. The perceived quality of services, the providers’ skills and the availability of medical equipment were the explanations of why some women preferred facility-based deliveries. |
| Seljeskog et al (37) | I. Some women preferred to give birth at home either with traditional attendants or alone  II. Most women preferred to receive childbirth care in a health facility with the assistance of skilled attendants. Majority of these women preferred to be assisted by a male nurse over a female nurse.  III. Most women preferred to have traditional postnatal care at home | I. A fear of having an operation was a key factor as to why women wanted to avoid a skilled childbirth in a hospital in favor of a traditional homebirth. Caesarean sections were considered negative and dangerous and linked with being lazy. Also, they feared longer hospital stays during childbirth due to time costs, including the costs of wages, time spent on travel, waiting time, and time spent by their caretaker. In consort, they were concerned about who would take care of house duties and children**.** Proximity, transportation and financial costs were other reasons why women preferred traditional homebirths with a TBA. Advice from trusted elders also convinced women to seek a traditional birth.  II. Another reason was that skilled attendants were considered competent for providing maternal care and managing complications. Skilled attendants were seen as safety measures to ensure a positive outcome for their birth. Male nurses were perceived to be more kind and personable than female nurses. Such positive experiences contrasted negative experiences with female nurses.  III. The traditional plants were herbs, which were deemed sufficient for the care of a healthy mother and baby; in their culture, they did not see the point of going to a facility for a postnatal checkup, with medical intervention only required when a postpartum complication arises. |
| Serizawa et al (38) | I. All women preferred to give birth at or near home with traditional attendants  II. All women preferred to have traditional postnatal care at home. | I. TBAs were favored as attendants because they ensured a home birth, which is the most comfortable, as it takes place in a familiar environment. Previous (positive) experiences in home birthing by TBAs convinced them that TBAs could offer a better quality of support than facility providers. This confidence also extended to cases of abnormal childbirth, as they trusted the TBAs’ experience as birth attendants for determining abnormal cases. A significant factor in preferring TBAs for home births was the inaccessibility and inconvenience of village midwives, who sometimes lived in other villages. In contrast, TBAs were said to be very accessible and convenient**,** even at night.  II. All participants believed that the mother and newborn baby must stay at home for 40 days after delivery because the mother and baby were vulnerable to witchcraft during this period (puerperal period). The belief in witchcraft and feelings of vulnerability during this period inhibited the women from obtaining facility-based postnatal care and encouraged them to stay at home. At home, they were also able to use traditional customs for protection against evil spirits that could harm them and their children. A key cultural reason for preferring TBAs was related to the practice of reinfibulation**,** which was a procedure performed in the postnatal period, largely for the sexual satisfaction of husbands, by a traditional surgeon in the village. TBAs supported this crucial custom, which was widely practiced among the village women. In contrast, village midwives largely forbade reinfibulation and did not support the traditional surgeons. Lastly, for time-related reasons, it was easier to stay home during the postpartum period. |
| Shiferaw et al (58) | I. Many preferred to have normal childbirth care and early traditional postnatal care at or near home with traditional attendants  II. A few women preferred to receive childbirth care in a health facility with the assistance of trained attendants. Some solely preferred trained health providers for complicated childbirth. | I. They perceived TBAs to be culturally acceptable and competent attendants. Mothers received needed support from their spouses and families’ presence in home deliveries with the assistance of TBAs. Traditional care is also preferred because TBAs attend to time-honored traditional practices rooted in the beliefs and cultures of the community; for example, laboring women were keen on having their abdomens massaged with butter as it was believed to facilitate smooth delivery. They were also allowed to take the placenta and bury it around their home after childbirth. Women who preferred a traditional childbirth indicated that some health professionals are not sensitive to their privacy and do not provide psychological support when they need it most. Having their naked body exposed to strangers discouraged them from using facility-based delivery services. Family members are also not allowed into most labor wards to provide needed support and comfort. As a result, the women valued the more supportive and comfortable care that they receive in traditional care from TBAs. They were also concerned about the quality of care at health facilities and lacked confidence and trust in health workers’ ability to deal with issues during the intrapartum period. Uneventful (positive) previous experiences further encouraged them to stay at home, with modern health care deemed unnecessary and as their second line of care or last resort. Elderly women in particular deemed institutional care as unnecessary due to past experiences in periods where there were no institutional healthcare services. In consort, based on past events, some women believed that when the first child is delivered at home successfully, nothing will happen to their babies in consecutive traditional childbirths at home.  II. A few women favored health facility services as they were critical of the care provided by TBAs due to safety concerns. One woman referred to how TBAs, contrary to health care providers, do not wear gloves, use clean equipment or use medications for delivery. TBAs were also accused of being careless in managing deliveries, thereby endangering the health of mothers and babies. In contrast, they believed facility deliveries were beneficial for mothers and babies. In situations of complicated labor and delivery, skilled care providers were deemed most competent in dealing with complications and ensuring a positive outcome. |
| Sialubanje et al (57) | I. Women prefer to give birth at or near home with traditional attendants  II. Some women preferred to receive childbirth care in a clinic facility. | I. Most women favored traditional childbirth care at or near home because of a low risk perception regarding their susceptibility to pregnancy and labor complications, based on their experience with childbirth. They believed their experience would help them recognize complications that may arise. Many women, mostly older participants, referred to past successful deliveries as a reason for wanting traditional homebirths. Other women preferred traditional homebirths due to the perceived harsh attitude and behavior of nurses towards laboring women. Some women, especially older women with children at home, chose unskilled homebirths because they did not want to be delivered by either a young nurse or a male staff at the clinic. TBAs on the other hand were female and often older. Pregnant women’s positive attitudes towards TBAs were reasons why most preferred traditional services provided by TBAs. TBAs provided assistance to women during labor in their communities. In addition, TBAs played an important role in providing health education and ANC services in designated health posts in the community. The flexibility of TBAs was also exemplified in the fact that they assisted deliveries in the women’s homes. They trusted TBAs to be immediately available when required to assist a laboring mother, whereas facilities were criticized for not having available providers. They were also familiar with TBAs**,** whereas facility-based health professionals were seen as strangers. They were free to discuss the progress of labor and their requirements without worries of being denigrated. They had confidence in the ability of TBAs because they had seen TBAs providing ANC services and conducting labor at the clinic. Contrary to nurses, TBAs were said to encourage the women during delivery and were more caring and compassionate as well. Several women, especially older women, believed that TBAs had enough skills and experience to assist with childbirth, including the ability to refer those with experienced complications to the clinic. The skills include cleansing the perineum, conducting the delivery, and detecting danger signs or complications.  II. Some women, younger women in particular, indicated a preference for facility-based deliveries due to their lack of experience in giving birth and fear of labor complications if they gave birth at home. |
| Sisay et al (59) | I. Majority of the women preferred to have ‘normal’ or uncomplicated childbirth at home. The preference shifted to skilled care in health facilities for complicated childbirth. | I. Homebirths without skilled care were believed to be the norm in the community and home the most comfortable place to deliver. On the other hand, facilities were only seen to be necessary for complicated childbirth requiring medical intervention at the nearest health facility. Some women were afraid of giving birth in facilities, even when experiencing complications such as prolonged labor. In particular, they were afraid of being left alone in a new environment as their relatives were not allowed to accompany them to the labor ward. Another reason was the belief that giving birth in a facility was associated with having bad fortunes |
| Thwala et al (53) | I. Majority of the women preferred to give birth at or near home with the assistance of traditional attendants  II. A few women preferred childbirth care at a hospital facility with skilled assistance. | I. The main explanations pertained to the tradition and habit of always having home births and feeling cared for and comfortable at home. Poor previous experiences with facility-based providers, who they felt treated them poorly, invigorated them to deliver at home. Traditional childbirths also provided women with personal control and freedom during the intrapartum period. Another factor for traditional childbirths over facility births was the fear of being alone and confined to a hospital bed during labor. The perceived lack of support they received while in labor, such as holding the laboring mother, also deterred them from giving birth at a facility. Some did not see the need for formally trained birth attendants, instead opting for their mothers- in-law, biological mothers or other women in the village who they felt treated them well and supported them throughout labor. Traditional homebirths were also favored because women were able to deliver in the position of their choice as guided by their instincts when giving birth. This enabled them to listen to their own bodies, rather than being forced to position themselves in certain ways at a facility. Such personal control and freedom meant they were free to make decisions during childbirth.  II. Some women felt safer giving birth at a hospital, as skilled attendants and medical interventions were available to deal with any complications. In consort, fear of experiencing complications at home under the care of untrained birth attendants also encouraged women to deliver in the hospital. There were some participants who simply felt that either formal or traditional care were appropriate for providing delivery services, as birth was seen as a natural occurrence regardless of the source of care. |
| Wilunda et al (66) | I. All women preferred to give birth at or near home with traditional attendants | I. Traditional care-takers offer a range of childbirth care services. One participant stated that TBAs are able to solve any problem that occurs, but also able to refer women to the health facility when their efforts to manage childbirth complications fail. Some women perceived facility staff as strangers and did not have as must trust in them as they did with their own family. Many women were better used to their own traditional delivery positions, including kneeling, squatting, sitting and lying on the side, which were not accepted in health facilities. Traditional care-takers were also more easily accessible and affordable than health professionals. Domestic chores and other family responsibilities, such as caring for children and preparing meals made homebirths the preferred option as women could care for their family. This was especially the case for women with infants at home because being admitted to a facility for delivery meant the children would be left alone at home with nobody to care for them. |
